# Comparative genomic and transcriptome analyses of pathotypes of *Xanthomonas citri* subsp. *citri* provide insights into mechanisms of bacterial virulence and host range

**DOI:** 10.1186/1471-2164-14-551

**Published:** 2013-08-14

**Authors:** Neha Jalan, Dibyendu Kumar, Maxuel O Andrade, Fahong Yu, Jeffrey B Jones, James H Graham, Frank F White, João C Setubal, Nian Wang

**Affiliations:** 1 Citrus Research and Education Center, Department of Microbiology and Cell Science, University of Florida, 700 Experiment Station Road, Lake Alfred, FL 33850, USA; 2 Waksman Genomics Core Facility, Rutgers University Busch Campus, Piscataway, NJ 08854, USA; 3 ICBR, University of Florida, Gainesville, FL 32611, USA; 4 Department of Plant Pathology, University of Florida, Gainesville, FL 32611, USA; 5 Department of Soil and Water Science, Citrus Research and Education Center, University of Florida, 700 Experiment Station Road, Lake Alfred, FL 33850, USA; 6 Department of Plant Pathology, Kansas State University, 4024 Throckmorton Hall, Manhattan, KS 66506, USA; 7 Departamento de Bioquímica, Instituto de Química, Universidade de São Paulo, São Paulo, SP 05508-000, Brazil; 8 Virginia Bioinformatics Institute, Virginia Polytechnic Institute and State University, Blacksburg, VA 24060-0477, USA

**Keywords:** *Xanthomonas citri*, Wellington strain, Citrus canker, HR, Virulence, Transcriptome, RNA-Seq

## Abstract

**Background:**

Citrus bacterial canker is a disease that has severe economic impact on citrus industries worldwide and is caused by a few species and pathotypes of *Xanthomonas. X. citri* subsp. *citri* strain 306 (XccA306) is a type A (Asiatic) strain with a wide host range, whereas its variant *X. citri* subsp. *citri* strain A^w^12879 (Xcaw12879, Wellington strain) is restricted to Mexican lime.

**Results:**

To characterize the mechanism for the differences in host range of XccA and Xcaw, the genome of Xcaw12879 that was completed recently was compared with XccA306 genome. Effectors *xopAF* and *avrGf1* are present in Xcaw12879, but were absent in XccA306. AvrGf1 was shown previously for Xcaw to cause hypersensitive response in Duncan grapefruit. Mutation analysis of *xopAF* indicates that the gene contributes to Xcaw growth in Mexican lime but does not contribute to the limited host range of Xcaw. RNA-Seq analysis was conducted to compare the expression profiles of Xcaw12879 and XccA306 in Nutrient Broth (NB) medium and XVM2 medium, which induces hrp gene expression. Two hundred ninety two and 281 genes showed differential expression in XVM2 compared to in NB for XccA306 and Xcaw12879, respectively. Twenty-five type 3 secretion system genes were up-regulated in XVM2 for both XccA and Xcaw. Among the 4,370 common genes of Xcaw12879 compared to XccA306, 603 genes in NB and 450 genes in XVM2 conditions were differentially regulated. Xcaw12879 showed higher protease activity than XccA306 whereas Xcaw12879 showed lower pectate lyase activity in comparison to XccA306.

**Conclusions:**

Comparative genomic analysis of XccA306 and Xcaw12879 identified strain specific genes. Our study indicated that AvrGf1 contributes to the host range limitation of Xcaw12879 whereas XopAF contributes to virulence. Transcriptome analyses of XccA306 and Xcaw12879 presented insights into the expression of the two closely related strains of *X. citri* subsp*. citri*. Virulence genes including genes encoding T3SS components and effectors are induced in XVM2 medium. Numerous genes with differential expression in Xcaw12879 and XccA306 were identified. This study provided the foundation to further characterize the mechanisms for virulence and host range of pathotypes of *X. citri* subsp*. citri*.

## Background

Members of the genus *Xanthomonas* are capable of infecting at least 124 monocot species and 268 dicot species and provide excellent case studies for understanding plant-microbe interactions [1]. Among the diseases caused by *Xanthomonas*, citrus canker caused by *X. citri* subsp*. citri* (Xcc) (syn. *X. axonopodis* pv. citri, *X. campestris* pv. citri, *X. citri* pv. citri) is an important disease that has severe economic impact on citrus industries worldwide. Asiatic (A) type strains are the most widespread and, hence, the most destructive form of citrus canker. The strains induce hyperplasic and hypertrophic (raised) lesions surrounded by oily or water-soaked margins and a yellow halo on leaves, stems, and fruits. Besides Xcc, a second species, *X. fuscans* subsp. *aurantifolii* (Xau), is also known to cause citrus canker with limited geographic distribution and limited host range. Type B strains of Xau are restricted to South America (Argentina, Uruguay and Paraguay) and cause canker on lemon (*C. limon*) and Mexican lime (*C. aurantifolia*). Type B strains can also be found on sweet orange (*C. sinensis*) and grapefruit (Citrus x *paradisi*) [2]. Type C strains of Xau are restricted to Brazil and cause canker only on Mexican lime [3].

Two variants of type A strains have also been identified. The variant designated A* was found in Southeast Asia in the 1990s infecting Mexican lime [4,5]. A second variant, designated as the “Wellington strain”, was isolated from Palm Beach County in southern Florida [4,6]. DNA hybridization analysis showed that Xcaw is more closely related to XccA and XccA* strains than to XauB and XauC strains [4]. Both Xcaw and XccA have similar symptoms and leaf populations on Mexican lime [7]. *X. citri* subsp. *citri* pathotype A^w^ (Xcaw) are pathogenic on Mexican lime and alemow (*C. macrophyla*) but not on grapefruit and sweet orange. The Xcaw strains cause a hypersensitive reaction (HR) in grapefruit [7]. The gene *avrGf1* was identified in Xcaw strain 12879, and mutation of *avrGf1* of Xcaw12879 rendered the mutant virulent on grapefruit, although the symptoms were much reduced as compared to symptoms due to strains of XccA306 [7]. A comprehensive understanding of the molecular mechanisms responsible for the differences in virulence and host range of Xcaw and XccA is lacking.

Comparative genomic analyses of xanthomonads have greatly facilitated our understanding of the virulence factors and host range determinants of different pathogens [8-10]. Comparative genomic analysis of *X. campestris* pv. campestris and XccA306 has been conducted previously to understand the mechanisms of different host range and pathogenic processes of the two *Xanthomonas* species, which have distinct host ranges [8]. Compared to Xcc, which infects citrus and causes citrus canker, *X. campestris* pv*.* campestris affects crucifers such as *Brassica* and causes black rot. Numerous species-specific genes have been identified which might explain the differing host specificities and pathogenic processes of the two pathogens. Comparative genomic analysis of XccA306 and *X. axonopodis* pv*.* citrumelo was also conducted recently [9]. *X. axonopodis* pv*.* citrumelo F1 is a nursery infecting strain and shows low virulence on citrus compared to that of XccA. Differences in gene contents, such as type III effectors (e.g., PthA), the type IV secretion system, and lipopolysaccharide synthesis were identified and may contribute to the differences in bacterial virulence and host range [9]. Furthermore, sequencing of XauB and XauC strains identified different virulence factors affecting host range of closely related species [10].

Here we conducted comparative genomic analysis of Xcaw12879 and the closely related strain XccA306 using a complete genome sequence of Xcaw12879 to understand the difference in virulence and host range. Recently, we have completed the genome sequencing of Xcaw12879 [11]. We further examined the transcriptomes of both XccA306 and Xcaw12879 by RNA-Seq in nutrient rich condition Nutrient Broth (NB) and in XVM2, which is known to induce hrp gene expression [12]. The comparative genomic and transcriptome analyses will provide the foundation to further characterize the mechanisms for virulence and host range of pathotypes of *X. citri* subsp*. citri*.

## Results

### Multi locus sequencing typing analysis

**Figure 1:**
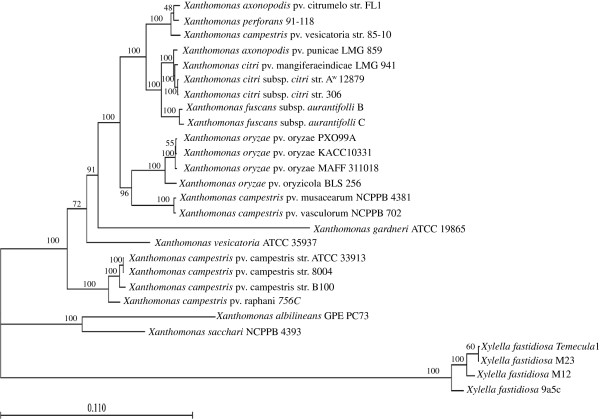
**Maximum likelihood phylogenetic tree of the genome of *Xanthomonas citri *subsp. *citri *A**^**w **^**12879 showing the relationship to other *Xanthomonads *and related species.** The tree was constructed using concatenated protein sequences of nine housekeeping genes (UvrD, SecA, CarA, RecA, GroEL, DnaK, AtpD, GyrB and InfB) aligned using Clustal W. Phylogenic tree from concatenated sequences was constructed in CLC Genomics workbench v6.0 using the Maximum likelihood method. The percentage of replicate trees in which the associated taxa clustered together in the bootstrap test (1000 replicates) are shown next to the branches. Horizontal scale bar (0.11) at the bottom represents number of amino-acid substitutions per site.

Multi locus sequence typing (MLST) based phylogenetic analysis was performed for Xcaw12879 and other *Xanthomonas* spp. using nine housekeeping genes (*uvrD, secA, carA, recA, groEL, dnaK, atpD, gyrB*, and *infB*) that are highly conserved in bacteria. The nine protein sequences were aligned and concatenated and then used to construct a maximum-likelihood phylogenetic tree (Figure 1). The results showed that Xcaw12879 is closely related to XccA306. Interestingly, these two citrus canker pathogens form a clade with *X. citri* pv. mangiferaeindicae LMG 941 and *X. axonopodis* pv. punicae LMG 859, which cause bacterial black spot in mango and bacterial leaf blight in pomegranates respectively. Both strains were isolated from India [13,14], a putative origin of XccA. Hence, it is possible that these pathogens have evolved from the same ancestor and evolved to adapt to different hosts. The XccA306 and Xcaw12879 strains share close relationship with the other two citrus canker causing bacteria XauB and XauC (Figure 1). The close relationship between XccA, Xcaw, XauB and XauC agrees with the genome-based phylogeny of the genus *Xanthomonas*[15].

### Chromosome organization and genome plasticity

**Figure 2:**
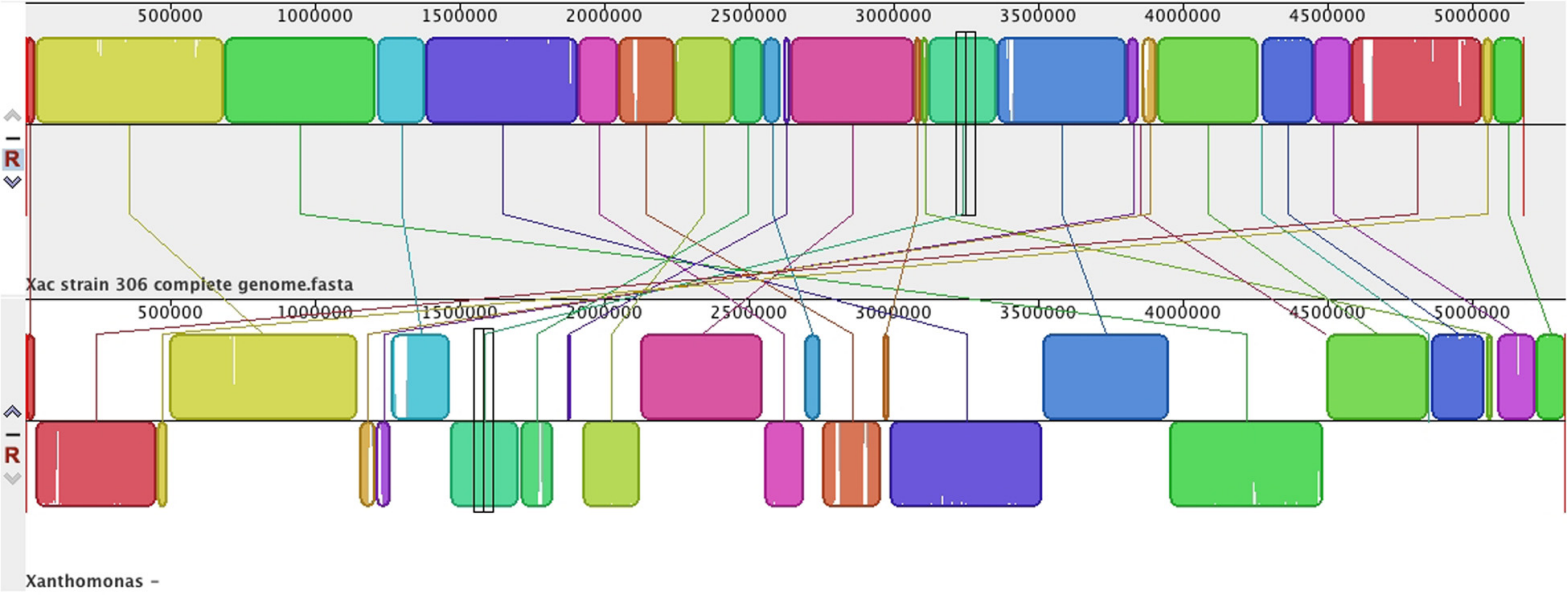
**MAUVE alignment of the genome sequences of *X. citri *subsp. *citri *str. 306 and *X. citri *subsp. *citri *A**^**w **^**12879.** Conserved and highly related regions are colored and low identity unique region are in white (colorless). The colored lines indicate translocations of the genome sections. Same colored blocks on opposite sides of the line indicate inversion.

Whole-genome alignment of Xcaw12879 to closely related XccA306 using MAUVE in progressive mode revealed numerous inversions and translocations (Figure 2). Most of the separated blocks in the alignment are associated with integrases and/or IS elements on at least one of their borders. The IS elements have been known to aid horizontal gene transfer and other genome rearrangements [16].

Xcaw12879 genome contains two plasmids pXcaw19 and pXcaw58 that are significantly different from the plasmids found in XccA306. Plasmid pXcaw19 sequence has no similarity with the plasmids of XccA306, whereas pXcaw58 is only about 35% similar to pXAC64. Plasmid pXcaw58 contains the *pthAw2* gene, a homolog of *pthA4*, which is capable of conferring the ability to cause canker-like symptoms [17]. However, the plasmid pXcaw58 does not contain the Vir like type IV secretion system genes found on pXAC64. The type IV secretion system has been shown to contribute to virulence in *X. campestris* pv. campestris strain 8004 [18] and absence of these genes from the plasmid could affect virulence of Xcaw12879 strain.

Three clustered regularly interspaced short palindromic repeats (CRISPRs) with short (21–47 bp) direct repeats interspaced with unrelated similarly sized non-repetitive sequences (spacers) are found in Xcaw12879 genome (Additional file 1). The CRISPR1 and CRISPR2 repeats are also present in XccA306. CRISPR2 and CRISPR3 from Xcaw12879 are identical except for a G at the beginning of CRISPR2, indicating that it might be a recent duplication. CRISPR is a bacterial immunity system that helps exclude foreign genetic elements. However the variability in Xcaw12879 and XccA306 suggests that the strains might have had dissimilar exposure to foreign genetic material as suggested in *X. oryzae*[19].

The TBLASTN analysis of all the proteins from Xcaw12879 and XccA306 revealed various gene clusters specific to each strain. Of the 4,760 proteins from Xcaw12879 and 4,603 (176 not annotated previously [8]) proteins from XccA306, 4,428 proteins are found to be orthologous using the cut-off e-value ≤ 10^-10^ and alignments >60% sequence identity, >60% query gene length. Among the 4,428 common proteins, 4,252 were annotated in XccA306 [8] whereas 176 are not annotated. Xcaw12879 has 332 proteins that are either non-orthologous to proteins from XccA306 or unique, whereas XccA306 has 175 such proteins.

The *hrp* and *hrc* genes encoding the type 3 secretion system (T3SS) in Xcaw12879 are homologous to the *hrp* and *hrc* genes found in XccA306. All the genes are found in similar order with the exception in gene annotation between *hrpF* and *hpaB*. The genome of XccA306 contains the annotated gene XAC0395 between the two, which is a hypothetical protein. The annotation in Xcaw12879 in the same region is on the opposite strand and contains *hpaI* (XCAW_00803) and *xopF1* (XCAW_00804/XCAW_00805) which may be nonfunctional due to a frameshift. The nucleotide sequences in both strains are the same and the differences in annotation were confirmed by BLAST similarity of the annotated genes in Xcaw12879 to other xanthomonads.

The T3SS translocates effector proteins into the plant cells. The effectors can either aid in nutrient acquisition and virulence or act as avirulence factors that trigger host immune response [20]. The type III effector genes in Xcaw12879 were predicted by BLAST analysis against the known T3SS effector database [http://www.xanthomonas.org]. Xcaw12879 contains thirty effector genes of which twenty-six overlap with XccA306 (except *pthA1*, *pthA2* and *pthA3*). Nineteen effectors are present in all four sequenced citrus canker causing variants compared (XccA, Xcaw, XauB, and XauC) and thus represent the core effector set for *Xanthomonas* that cause citrus canker. It is noteworthy that Escalon et al. [21] define a ‘common repertoire’ of 26 T3S effector genes present in 55 Xcc strains from several locations around the world. They did not use data from XauB and XauC in compiling this common repertoire which explains why 26 T3S effectors were identified previously [21] whereas we only identified 19 common T3S effectors. The effector genes *avrBs2, xopK, xopL, xopQ, xopR, xopX* and *xopZ* are found in all other sequenced *Xanthomonas* genomes and hence these seven genes might be a core set of effectors required for phytopathogenicity as suggested by Moreira *et al.*[10]. Twelve effector genes (*xopA, xopE1, xopE3, pthA4* or its functional homologs*, xopI, xopV, xopAD, xopAI, xopAK, xopAP, hpaA*, and *hrpW*) are present in all four citrus canker causing variants (Xcaw, XccA, XauB and XauC). Of the twelve effector genes, *xopE3* and *xopAI* are present in Xcaw12879 albeit in different locations as compared to the potential genomic island in the other three strains causing citrus canker. However they may play a role in citrus canker as suggested by Moreira *et al.*[10]. Two effector genes *avrGf1* and *xopAF* were identified in Xcaw, XauB and XauC but were not present in XccA306 genome (Table 1).

**Table 1:** **Effector repertoire of *X. citri *subsp. *citri *A**^**w **^**12879 (Xcaw12879), *X. citri *subsp*. citri *str. 306 (XccA306)*, X. fuscans *subsp. *aurantifolii *str. ICPB 11122 (XauB) and *X. fuscans *subsp. *aurantifolii *str. ICPB 10535 (XauC)**

**Effector class**	**Xcaw12879**	**XccA306**	**XauB**	**XauC**	**Pfam domains**	**References**
AvrBs2	XCAW_00465	XAC0076	XAUB_16770	XAUC_23650	Glycerophosphoryl diester phosphodiesterase	[22]
PthA (AvrBs3, TAL)	XCAW_b00018 (PthAw1)	XACa0022 (PthA1)	XAUB_40130	XAUC_22430	Transcriptional activator, nuclear localization	[23]
		XACa0039 (PthA2)		XAUC_24060		
	XCAW_b00026 (pthAw2)	XACb0015 (PthA3)	XAUB_28490	XAUC_09900		
		XACb0065 (PthA4)		XAUC_43080		
XopA (Hpa1/HpaG)	XCAW_00826	XAC0416	XAUB_19280	XAUC_43660	-	[24]
XopE1 (AvrXacE1)	XCAW_00686	XAC0286	XAUB_37010	XAUC_37580	Putative transglutaminase	[25]
XopE3 (AvrXacE2)	XCAW_03515	XAC3224	XAUB_14680	XAUC_00040	Putative transglutaminase	[26]
XopF2	XCAW_0138Ψ	XAC2785 Ψ	XAUB_07540Ψ	XAUC_21000Ψ	-	[27]
XopI	XCAW_03828	XAC0754	XAUB_39080	XAUC_07100	F-box protein	[28]
XopK	XCAW_03372	XAC3085	XAUB_34090	XAUC_12520	-	[29]
XopL	XCAW_03376	XAC3090	XAUB_34130	XAUC_02900/XAUC_12488Ψ	LRR protein	[30]
XopQ	XCAW_04706	XAC4333	XAUB_10220	XAUC_14670	Inosine uridine nucleoside N-ribohydrolase	[27]
XopR	XCAW_00677	XAC0277	XAUB_36920	XAUC_37490	-	[29]
XopV	XCAW_03980	XAC0601	XAUB_23140	XAUC_21260	-	[29]
XopX	XCAW_00956	XAC0543	XAUB_14760	XAUC_20690	-	[31]
XopZ1	XCAW_01815	XAC2009	XAUB_11532/XAUB_13710Ψ	XAUC_25915	-	[26]
XopAD	XCAW_00082	XAC4213	XAUB_02510	XAUC_34870	SKWP repeat protein	[32,33]
XopAI	XCAW_01099	XAC3230	XAUB_26830	XAUC_23780	Putative ADP- ribosyltransferase	[34]
XopAK	XCAW_04369	XAC3666	XAUB_02580	XAUC_32490	-	[33]
XopAP	XCAW_03269	XAC2990	XAUB_13980	XAUC_08760	-	[35]
HpaA	XCAW_00810	XAC0400	XAUB_19430	XAUC_19990	T3S control protein	[36]
HrpW (PopW)	XCAW_03200	XAC2922	XAUB_19460	XAUC_20020	Pectate Lyase	[37]
XopAQ	XCAW_03514	Not annotated*	Not annotated**	-	-	[35]
XopE2 (AvrXacE3, AvrXccE1)	XCAW_03520	XACb0011	XAUB_31660	-	Putative transglutaminase	[25]
XopN	XCAW_01387	XAC2786	XAUB_07520	-	ARM/HEAT repeat	[38]
XopP	XCAW_01310	XAC1208	XAUB_06720	-	-	[27]
XopAE (HpaF/HpaG)	XCAW_00801	XAC0393	XAUB_19500	-	LRR protein	[39]
XopC2	XCAW_01311Ψ	XAC1209/XAC1210Ψ			Haloacid dehalogenase-like hydrolase	[39]
XopAF (AvrXv3)	XCAW_b00003	-	XAUB_02310	XAUC_00300	-	[40]
XopAG (AvrGf1/AvrGf2)	XCAW_00608	-	XAUB_03570 Ψ	XAUC_04910	-	[7]
XopF1(Hpa4)	XCAW_00804/XCAW_00805Ψ	-		XAUC_31730Ψ		[30]
XopB	-	-	XAUB_09070/XAUB_14842 Ψ	XAUC_00260	-	[41]
XopE4	-	-	XAUB_23330	XAUC_31730	Putative transglutaminase	[10]
XopJ1	-	-	XAUB_20830	XAUC_08850	C55-family cysteine protease or Ser/Thr acetyltransferase	[27]

Ψ Inactive/Pseudogene.

* Located between XAC3223 and XAC3224 from 3,797,415 bp to 3,797,702 bp.

** Located between XAUB_14670 & XAUB_14680 on NZ_ACPX01000163 from 23,262 bp to 23,549 bp.

**Figure 3:**
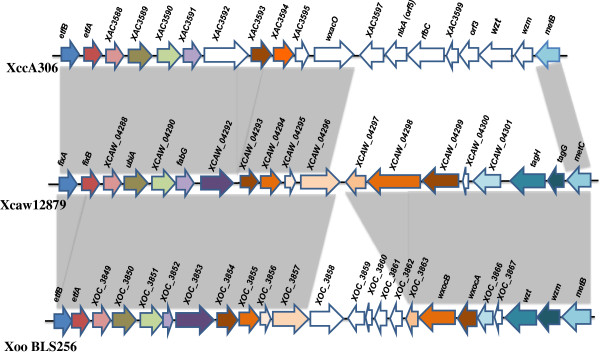
**Comparison of the LPS gene clusters of *X. citri *subsp. *citri *str. 306, *X. citri *subsp. *citri *A**^**w **^**12879 and *X. oryzae *pv. oryzicola str. BLS256.** Conserved and highly related genes (over 80% identity) are colored and syntenic regions between the bacteria are shaded in grey (over 50% identity).

Multiple genes clustered into ten groups were identified in Xcaw12879 but not in XccA306 (Table 2). Many genes of these clusters present in Xcaw12879 but not in XccA306 have homologs in other *Xanthomonas* species. All these regions contain transposase, integrase or phage related genes indicating possible acquisition by horizontal gene transfer. An interesting difference noted in the above-mentioned regions is in cluster 5, which encodes for lipopolysaccharide (LPS) biosynthetic pathway. Interestingly, the LPS cluster in Xcaw12879 contains regions orthologous to both XccA306 and *X. oryzae* pv. oryzicola BLS256 as shown in Figure 3, which indicates that there has been horizontal gene transfer. Cluster 4 is almost 100 kb long and parts of cluster 4 are syntenic with regions from *X. campestris* pv*.* campestris 8004, a black spot pathogen of cabbage (Table 2). A MUMmer comparison between cluster 4 and *X. campestris* pv*.* campestris 8004 shows high synteny (Additional file 2). Three transcriptional regulators (XCAW_01037, XCAW_01129, XCAW_01131) and one two-component system (TCS) sensor kinase (XCAW_01148) and its response regulator (XCAW_01150) are present in Xcaw12879, but are absent in XccA306.

**Table 2:** Gene clusters present in Xcaw12879 but absent in XccA306

**Cluster number**	**Locus tag**	**Homologs in other genomes**	**Function**
1	XCAW_01029 to XCAW_01069		hypothetical proteins, RhsA family protein, transcriptional regulator, integrase, adenine specific DNA methylase, type III restriction enzyme: res subunit, ATP dependent exoDNAse, thermonulease
2	XCAW_01117 to XCAW_01151	Some present in *X*. *campestris* pv. campestris ATCC 33913	transcriptional regulator, phage-related tail proteins, TCS response sensor and regulator, chitinase, Zn peptidase, transcriptional repressor, protein -glutamate methylesterase
3	XCAW_01571 to XCAW_01582	Some present in *X*. *campestris* pv. campestris str. 8004	phage related proteins, hypothetical protein
4	XCAW_01620 to XCAW_01726	Homologous to *Acidovorax* sp. JS42 and *X*. *campestris* pv. campestris str. 8004	transposases, hypothetical proteins, type II restriction enzyme: methylase subunit, phage related regulatory proteins, chromosome partitioning related protein, soluble lytic murein transglycosylase, VirB6 protein
5	XCAW_04292 to XCAW_04303	Homologous to *X*. *oryzae* pv. oryzicola BLS256	lipopolysaccharide biosynthesis genes
6	XCAW_04482 to XCAW_04496		transposases, hypothetical proteins, transcriptional repressor, polymerase V subunit
7	XCAW_04519 to XCAW_04545	Homologous to *X*. *oryzae* pv. oryzae PXO99A	phage related proteins, transcriptional regulator, transposases, hypothetical proteins
8	XCAW_a00001 to XCAW_a00017		Plasmid partition protein, conjugal transfer protein, hypothetical proteins
9	XCAW_b00002 to XACW_b00018		Transposases, plasmid stability proteins, avirulence protein, hypothetical proteins
10	XCAW_b00048 to XACW_b00056		Plasmid mobilization proteins, transposases, hypothetical proteins

### PthA and homologs

All the citrus canker causing variants (XccA, Xcaw, XccA*, XauB, and XauC) contain PthA or its functional homologs. Thus, PthA or its functional homologs is likely the pathogenicity determinant of citrus canker pathogen as suggested in a previous study [17] that linked the strains of *Xanthomonas* with different host range together. Al-Saadi et al. [17] have shown that all the variants carry one *pthA* homolog with 17.5 repeats which determines pathogenicity on citrus and triggers immunity in various other plant species [42]. The *avrBs3*/*pthA* family of effectors includes various *pth* genes but only PthA [42] is known to induce canker. The functional homolog of this gene in XccA strain 306 is *pthA4*, which also has three other paralogs on its two plasmids (Table 1). We found two homologs *pthAw1* and *pthAw2* in Xcaw12879 genome, both located on plasmid pXcaw58. The *pthAw2* gene is 99% identical to *pthA4* from XccA and also to *pthAw* sequenced from another Wellington strain 0053 that is able to complement a knockout mutant of *pthA* in XccA strain 3213 [17], indicating that PthAw2 is the functional homolog of *pthA* in Xcaw. PthAw2 has the same repeat number (17.5) as the functional homologs PthA4, PthB and PthC from the three respective citrus canker causing strains XccA, XauB, and XauC [10]. The other homolog PthAw1 in Xcaw has 18.5 tandem repeats, which is different from PthA homologs found in XccA that have either 15.5 or 16.5 tandem repeats. The AvrBs3/PthA family of effectors are known as transcription activator-like (TAL) effectors since they reprogram host cells by specifically binding to the promoters of plant genes recognized by the central domain of tandem repeats [43]. Comparing the DNA binding TAL effector codes for PthA from XccA as predicted by Boch et al. [44] to PthAw indicate that the codes for PthA4 and PthAw2 are quite divergent (Additional file 3). Al-Saadi et al. [17] predicted that the well conserved sequence of the 17^th^ repeat in functional PthA might be important for pathogenicity on citrus, and this sequence is present in PthAw2. The rest of PthAw2 sequence however potentially encodes a DNA binding code that is only about 79% similar to the one encoded by PthA4 of XccA306 (Additional file 3). This may result in recognition of different target genes in host plant or differences in strength of induction of plant genes and thus affect virulence of Xcaw and XccA.

### Pathogenicity and growth assays

All three host limited strains of *Xanthomonas* affecting citrus, Xcaw, XauB and XauC had *avrGf1* and *xopAF* genes in their genomes. The gene *avrGf1* has been previously studied in Xcaw and is known to be responsible for HR in grapefruit [7]. However its effect on other varieties of citrus such as sweet orange is unknown. Also, since *xopAF* is the other putative effector gene, its effect on host limitation was further characterized by pathogenicity and growth assays of XcawΔ*xopAF* and XcawΔ*avrGf1*Δ*xopAF*.

**Figure 4:**
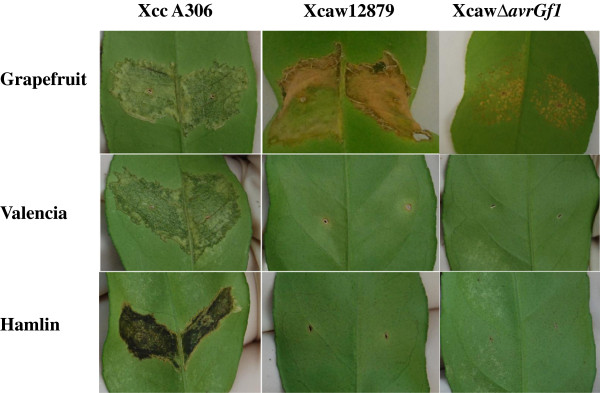
**Pathogenicity assay *in planta*.** Inoculation by pressure infiltration of *X. citri* subsp. *citri* str. 306, *X. citri* subsp. *citri* str. A^w^12879 and *X. citri* subsp. *citri* str. A^w^Δ*avrGf1* mutant on young Duncan grapefruit, Valencia and Hamlin leaves. The culture concentration of 10^8^ cfu/ml was used for inoculation and leaves were photographed after 10 days of incubation. XccA306 infects all three citrus varieties; Xcaw12879 shows hypersensitive reaction only on grapefruit. XcawΔ*avrGf1* mutant shows reduced symptoms as compared to XccA306 on grapefruit and no symptoms on Valencia and Hamlin.

Pathogenicity assays indicated that Xcaw12879 did not elicit any reaction on Valencia or Hamlin oranges at our test conditions while wild type XccA306 caused necrotic raised lesions, typical of citrus canker on the leaves at a high bacterial inoculation concentration of 10^8^ cfu/ml (Figure 4). Xcaw12879 showed a HR on grapefruit leaves that was abolished by deleting *avrGf1* gene (XcawΔavrGf1), however the growth of the mutant was visibly reduced compared to XccA306 strain. XcawΔ*avrGf1* did not show any symptoms or reaction on either Valencia or Hamlin (Figure 4).

**Figure 5:**
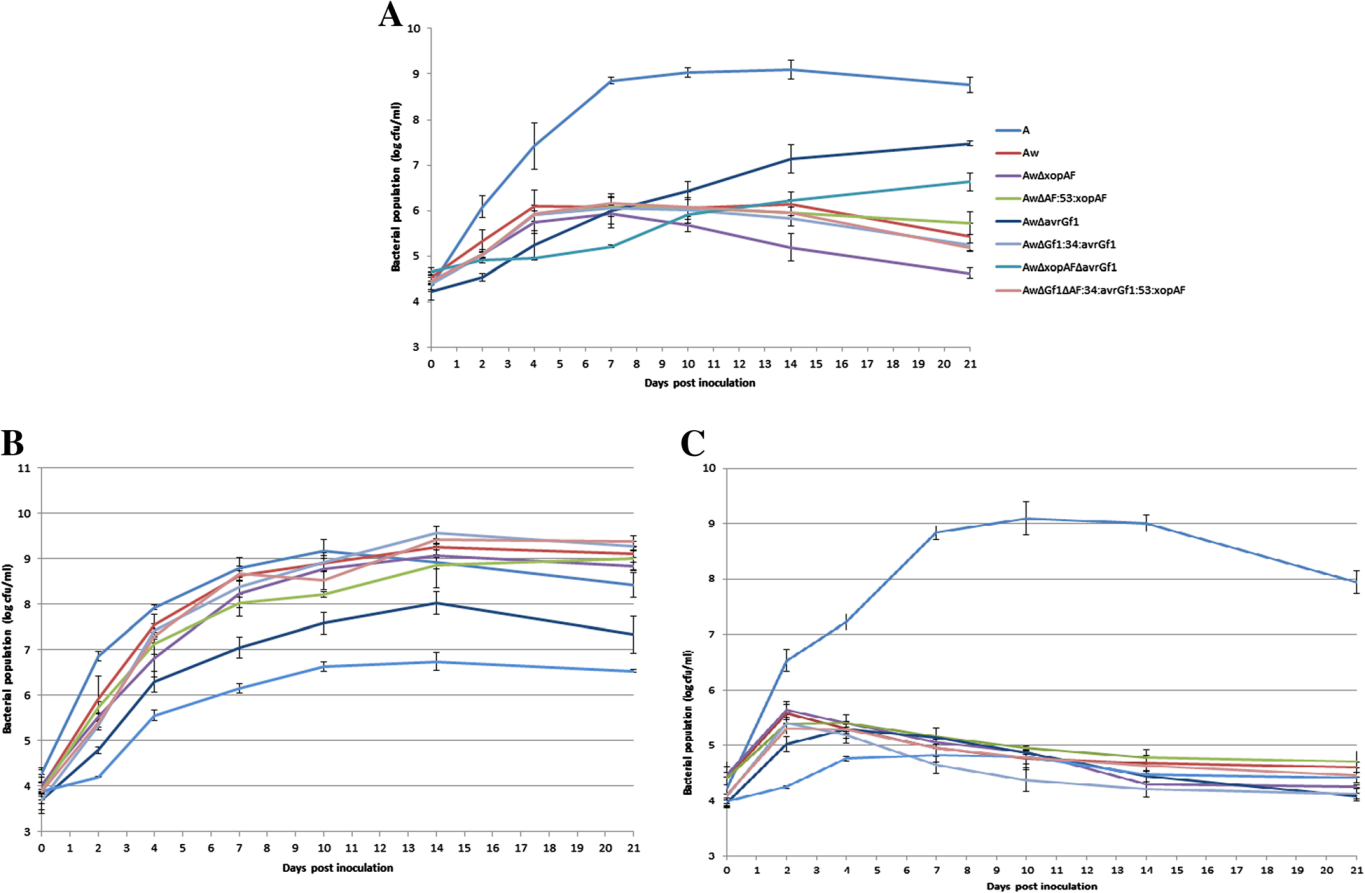
**XopAF contributes to the growth of Xcaw12879 strain *in planta*.** XccA306 (A), Xcaw12879 (Aw), Xcaw12879Δ*xopAF* (AwΔxopAF), Xcaw12879Δ*xopAF-*53:*xopAF* (AwΔAF:53:xopAF, complement strain), Xcaw12879Δ*avrGf1* (AwΔavrGf1), Xcaw12879Δ*avrGf1*-34:*avrGf1* (AwΔGf1:34:avrGf1, complement strain), Xcaw12879Δ*xopAF*Δ*avrGf1* (AwΔxopAFΔavrGf1) and Xacw12879 Δ*xopAF*Δ*avrGf1*-34:*avrGf1*-53:*xopAF* (AwΔGf1ΔAF:34:avrGf1:53:xopAF, complement strain) were inoculated at approximately 10^6^ cfu/ml concentration into **A**. Duncan grapefruit, **B**. Mexican lime and **C**. Valencia sweet orange leaves using a needleless syringe. Bacterial cells from the inoculated leaves were recovered at different time-points, diluted and counted to plot the growth curve. The values at each time point represent means of three replicates. Means ± SD are plotted.

To check whether mutation of *xopAF* affects Xcaw12879 growth *in planta*, the wild-type strains of XccA306 and Xcaw12879, XcawΔ*xopAF*, XcawΔ*xopAF*-53:*xopAF* (complementary strain), XcawΔ*avrGf1* and XcawΔ*xopAF*Δ*avrGf1* mutant strains were inoculated into grapefruit, Mexican lime and Valencia leaves. As shown in Figure 5A, the population of Xcaw12879 was much lower compared to XccA306 in grapefruit. This population of XcawΔ*avrGf1* was increased compared to the wild type Xcaw12879 and XcawΔ*avrGf1* caused symptoms on grapefruit. However, the populations of XcawΔ*xopAF* and XcawΔ*xopAF*Δ*avrGf1* mutants were one order magnitude lower than that of Xcaw12879 and XcawΔ*avrGf1* respectively, indicating that mutation of *xopAF* gene decreased the growth of Xcaw12879 *in planta*. A similar trend was observed in Mexican lime where the populations of *xopAF* single and *xopAF, avrGf1* double mutants were lower compared to Xcaw12879 and XcawΔ*avrGf1* respectively (Figure 5B). The growth of XcawΔ*xopAF* in grapefruit and Mexican lime was restored to similar levels as Xcaw12879 by the complementation (Figure 5). No significant changes were observed in Valencia leaves as neither Xcaw12879 nor any of its mutants grew well in the sweet orange variety as compared to XccA306 (Figure 5C).

### Transcriptome analyses of Xcaw12879 and XccA306 under nutrient rich (NB) and *hrp* gene expression inducing (XVM2) conditions

To determine the differential gene expression amongst the strains of *X. citri* subsp. *citri*, we grew Xcaw12879 and XccA306 under nutrient rich condition in Nutrient Broth (NB) and in XVM2 [12]. Three biological replicates of the strains were used for RNA-Seq. Over 45 million reads were obtained on average for each sample. After trimming and mapping, approximately 96% of the reads were mapped to the genomes (data not shown) indicating that RNA-Seq provides high quality reads suitable for *Xanthomonas* transcriptomics. Of all the reads, over 6.5 to 14 million reads could be mapped from each sample to mRNA specifically (Additional file 4). This gave an enrichment of mRNA from 11.3% up to 28.5% for each sample. It has been suggested that 5–10 million non-rRNA fragments enable profiling of the vast majority of transcriptional activity in diverse species including *E. coli* grown under diverse culture conditions [45]. It was also found that when RNA-Seq data from biological replicates is available, differential expression of numerous genes can be detected with high statistical significance even when the number of fragments per sample is reduced to 2–3 million [45]. Thus our RNA-Seq data is likely sufficient for the transcriptome analysis of XccA306 and Xcaw12879.

To quantify the expression of each gene, the reads aligned to each gene were pooled and normalized for gene size by calculating the Reads Per Kb per Million reads (RPKM) values. The values for each gene from all the replicates were further quantile normalized to test them statistically. The resulting values were log_2_ transformed and *t*-test was performed on these expression values to compare differential gene expression (DGE) between XccA306 and Xcaw12879 under the same growth conditions or between the same strains in NB or XVM2 growth conditions. High correlation was observed between differential expression values of biological replicates (Additional file 5), signifying that the method was reproducible. Principal component analysis indicates that the biological replicates of XccA formed a separate cluster from Xcaw in both growth conditions (Additional file 6).

qRT-PCR was used to validate the RNA-Seq data. Eight genes were chosen (Additional file 7) that were differentially expressed in Xcaw as compared to XccA under both NB and XVM2 growth conditions to compare data obtained from the two methods. The resulting transcriptional ratio from qRT-PCR analysis was log_2_ transformed to compare with the DGE values obtained by RNA-Seq (Additional file 8). Although the scale of fold changes between the two techniques is different, high correlation coefficient of 0.87 verifies that the general trend of gene expression is consistent for both data sets.

We studied the expression profile of Xcc strains in XVM2 as compared to NB. At the cut-off of │fold change│ = 3, FDR < 0.05, 292 genes showed differential expression (173 up-regulated and 119 down-regulated in XVM2 compared to NB) in XccA (Additional file 9) and 281 genes (129 up-regulated and 152 down-regulated in XVM2 compared to NB) for Xcaw (Additional file 10). The entire T3SS cluster consisting of twenty-five genes except one gene (XAC0395) was up-regulated in XVM2 for both XccA and Xcaw strains (Additional files 9 and 10). Among all the effectors, sixteen were induced for XccA whereas nineteen effectors were overexpressed for Xcaw in XVM2 compared to in NB. As identified in this study, the effector genes *avrBs2, xopA, xopE1, xopE3, xopI, xopX, xopZ1, xopAD, xopAP, xopAQ, hpaA, xopN* and *xopP* were up-regulated in XVM2 in both strains, while *pthA1*, *pthA2*, *avrXacE3* and *xopK* were induced only in XccA and *xopL, xopR, xopAI, xopAK, xopAF* and *xopAG* only in Xcaw strain.

The 11-gene *xps* cluster encodes for type 2 secretion system (T2SS) in *Xanthomonas* secreting various enzymes including pectate lyase, cellulase, and xylanase. The *xps* genes were down-regulated in XVM2 as compared to in NB for Xcaw, with *xpsE* being the most significantly down-regulated. For XccA, the *xps* genes were not down-regulated. Besides the T2SS genes, at least 22 genes encoding T2SS substrates in XccA were overexpressed in XVM2 as compared to only 12 in Xcaw. To the contrary 11 genes for Xcaw and 8 for XccA were down-regulated in XVM2 compared to in NB (Additional files 9 and 10).

Our analysis showed that all the flagella biosynthesis genes encoded by *flg* and *fli*, motility by *mot* and chemotaxis by *mcp*, *che* and *tsr* were repressed in XVM2 for XccA and Xcaw except *cheY* (XAC3284 in XccA and XCAW_03412 in Xcaw) and *tar* (XCAW_03417, XCAW_04009 and XCAW_02497). The genes encoding LPS were down-regulated in both Xcaw and XccA, whereas the xanthan gum (EPS) genes were overexpressed in both except *gumP* in XccA. A few genes encoding outer membrane proteins, which are involved in adhesion, including *ompW*, *blc* and *hms* were up-regulated in XVM2 as compared to in NB for both strains while *xadA* and *yapH* were induced in XccA but down-regulated in Xcaw. The Type IV pili genes encoded by *pil* and *fim* genes except *pilB* and filamentous haemagglutinin related genes (*fhaB*, *XAC1816*) were down-regulated in both Xcaw and XccA (Additional files 9 and 10).

**Figure 6:**
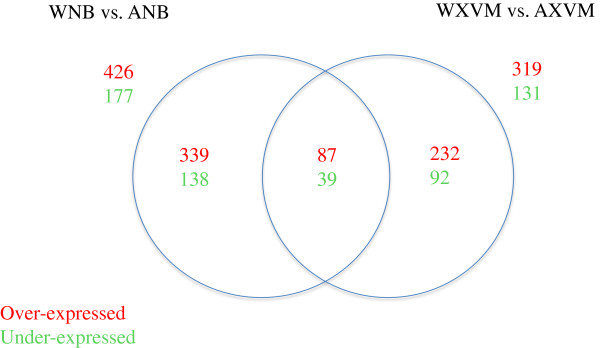
**Number of differentially expressed genes when comparing expression of common genes in *X. citri *subsp. *citri *str. A**^**w **^**12879 to *X. citri *subsp. *citri *str. 306 in NB and XVM2 growth conditions.** Gene expression of orthologous genes between Xcaw (W) and XccA (A) was compared when grown in Nutrient broth (NB, nutrient rich medium) and XVM2 (XVM, hrp inducing medium).

In order to further understand the molecular mechanisms determining the differences in virulence and host range of Xcaw and XccA, we compared the expression profile of common genes of Xcaw and XccA. When expression of orthologous genes in Xcaw was compared to XccA, 603 genes (426 overexpressed and 177 down-regulated) in NB (Additional file 11) and 450 genes (319 overexpressed and 131 down-regulated) genes in XVM2 (Additional file 12) conditions were significantly differentially regulated at cut-off value of │fold change│ = 3 and FDR < 0.05. On comparing the differentially expressed genes in both conditions, 126 genes were differentially regulated in Xcaw as compared to XccA, irrespective of the growth conditions (Figure 6). Of these 87 were overexpressed in Xcaw and 39 genes were repressed as compared to XccA (Additional file 13). Of the 87 genes overexpressed in Xcaw, 35 were virulence-related genes including *hrpX*, *hrpG*, *phoP*-*phoQ* regulatory genes, and T2SS substrate genes (XAC2537, XAC2763, XAC2999, XAC4004) (Additional file 13). Of the 39 genes overexpressed in XccA, 21 were virulence-related genes including cellulase genes (XAC0028, XAC0029 and *engXCA*), reactive oxygen species scavenging enzyme genes, e.g., superoxide dismutase gene *sodC2*, and genes encoding heat shock protein GrpE and heat stress protein Muc.

**Figure 7:**
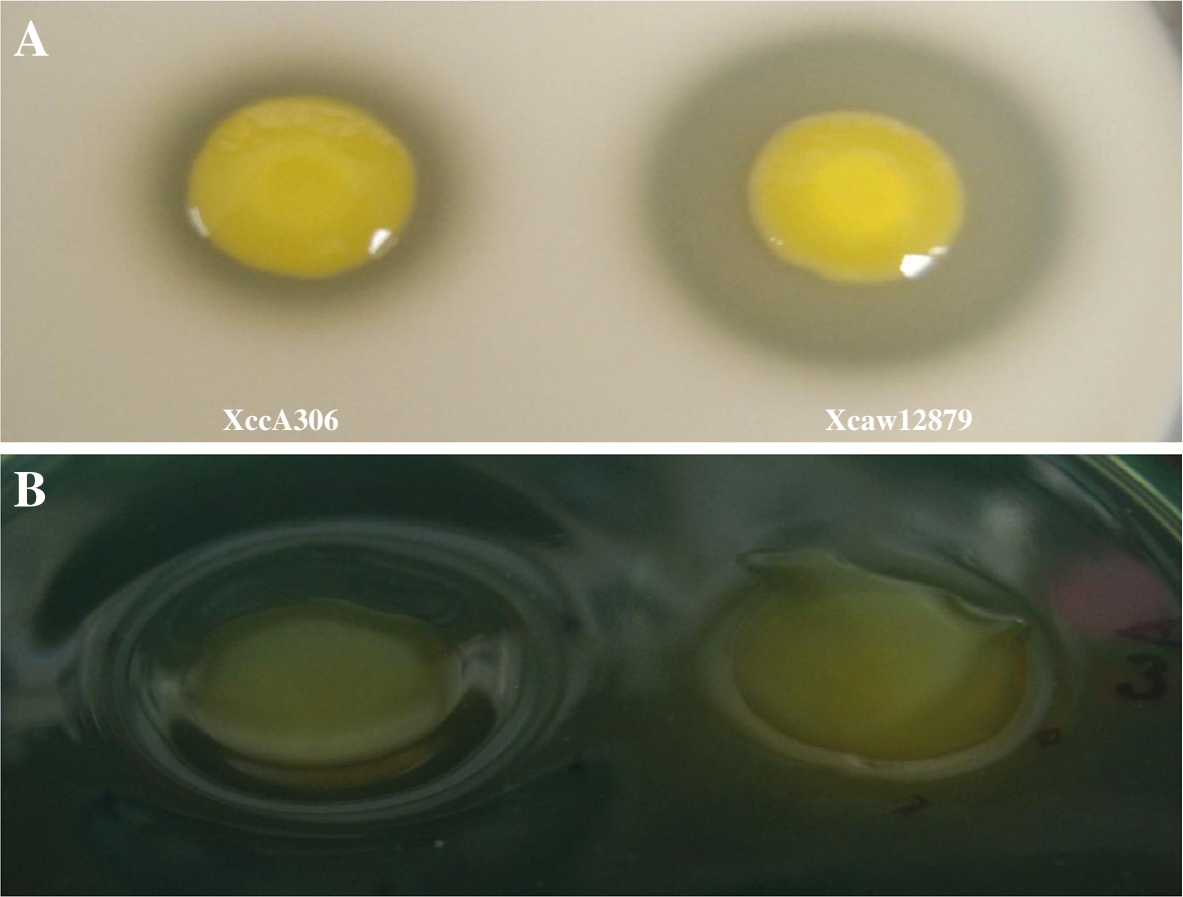
**Protease and Pectate lyase activity of *X. citri *subsp. *citri *str. 306, and *X. citri *subsp. *citri *str. A**^**w **^**12879. (A)** Protease activity was tested by inoculating 1 μl culture on 10% milk agar plates at 28°C for 6 days. Zone of clearance was used as the measure of protease activity. **(B)** Pectate lyase activity was tested by inoculating 1 μl culture on Hildebrand’s agar medium at 28°C for 6 days. More pitting can be seen on medium at pH 8.5 for XccA strain compared to Xcaw.

Since the gene expression of T2SS substrate genes was different, we compared the protease and pectate lyase activities of XccA306 and Xcaw12879. Xcaw12879 showed higher protease activity than XccA306 (Figure 7A). Xcaw12879 showed lower pectate lyase activity compared to XccA306 (Figure 7B).

## Discussion

Comparative analysis of Xcaw12879 and XccA306 identified multiple strain-specific genes that might contribute to the differences in virulence and host range. Among the genes present in Xcaw12879, but absent in XccA306, two effector genes *xopAG (avrGf1)* and *xopAF* were identified in Xcaw, XauB and XauC but were not present in XccA306 genome (Table 1). The presence of these effectors in limited host range strains causing citrus canker and not in the broader host range XccA306 makes them prime candidates for effectors that could affect host specificity. Importantly, the role of *xopAG* (*avrGf1*) in limiting the host range of Xcaw has been confirmed previously [7]. The *xopAG* gene belongs to the *avrGf1* family and has been shown to trigger HR in grapefruit [7]. AvrGf1 in Xcaw shows only about 45% identity to its homolog XAUC_04910 in XauC whereas the homolog XAUB_03570 in XauB is interrupted by a transposon and might be non-functional, which probably contributes to the broader host range of XauB compared to Xcaw and XauC [2,3]. When the mutant XcawΔ*xopAG* was inoculated in grapefruit it caused typical canker like symptoms instead of HR, but the symptoms were visibly reduced [7]. Also, XcawΔ*avrGf1* does not cause disease on sweet orange (Valencia and Hamlin) as shown in Figure 4, indicating that there are other host limiting factors in the Xcaw12879 genome or other virulence factors are required for XccA306 to infect different hosts. Another candidate gene, which might contribute to host specificity, is *xopAF*, which belongs to *avrXv3* family and is located on the plasmid pXcaw58 in Xcaw12879. Homologs of *xopAF*, XAUB_02310 and XAUC_00300 are found in XauB and XauC but not in XccA306 (Table 1). Thus, we initially hypothesized that XopAF may contribute to restricting host range of Xcaw12879, XauB, and XauC to limited varieties of citrus as compared to XccA306. Additionally, an *xopAF* homolog *avrXv3* from *X. campestris* pv. vesicatoria is known to induce HR in tomato line Hawaii 7981 and pepper plants [40]. The same work also ascertained that the gene was plant inducible and regulated by the *hrp* regulatory system. The C terminal region of the protein encodes for a putative transcription activator domain indicating that it might interact with plant host genes. In this study we found that *xopAF* mutant and *xopAF avrGF1* double mutant both have lower growth *in planta* as compared to Xcaw12879 and *avrGF1* single mutant respectively (Figure 5). Mutation of *xopAF* did not make Xcaw12879 strain pathogenic in sweet orange Valencia. Instead, mutation of *xopAF* slowed the growth of the pathogen in grapefruit and Mexican lime, which was restored by complementation, indicating that XopAF is important for bacterial growth *in planta*. In addition to the effectors documented above, other effectors that differ in their presence are *xopAQ*, *xopE2*, *xopN*, *xopP* and *xopAE*, present in Xcaw12879, XccA306 and XauB but not in XauC strain. Also *xopB*, *xopE4* and *xopJ1* are present in both XauB and XauC but missing from XccA306 and Xcaw12879. How these effectors contribute to virulence and host range of XccA, Xcaw, XauB, and XauC requires further investigation.

Other gene content differences between Xcaw12879 and XccA306 include differences in LPS cluster (Figure 3), phage related genes with Xcaw containing XCAW_1134 to XCAW_1142, XACW_4520 to XCAW_4227 whereas XccA exclusively includes XAC1063, XAC2628, and Type IV secretion system and a plant-like natriuretic peptide (PNP) encoding gene (XAC2654). Interestingly, all the genes in LPS cluster from Xcaw12879 show high similarity with LPS region from rice pathogen *X. oryzae* pv. oryzicola BLS256, whereas only approximately half the cluster is syntenic to XccA306 LPS cluster (Figure 3). This suggests that HGT has probably resulted in a hybrid LPS cluster in Xcaw12879 similar to *X. oryzae* pv. oryzicola BLS256 [46]. LPS, phage related proteins, type IV secretion system and PNP have been reported to play certain roles in virulence [18,47-51]. How they contribute to the difference of Xcaw and XccA in virulence and host range remains to be investigated experimentally.

Virulence related genes were differentially regulated in XVM2 as compared to NB for both Xcaw12879 and XccA306. In XccA306 (Additional file 9), fifty-nine virulence related genes were induced and thirty-eight genes were repressed in XVM2 compared to NB. In Xcaw12879 (Additional file 10), forty virulence related genes were induced and twenty-four genes were repressed in XVM2 compared to NB. The induction of the virulence genes in XVM2 condition compared to nutrient rich NB is supported by a previous study [52]. In the previous study, only 279 genes of XccA potentially associated with pathogenicity and virulence were tested and 31 genes were up-regulated in XVM2, while only 7 genes were repressed. In our study, we further expanded the previous study by including all genes of XccA and provided a comprehensive picture of *Xanthomonas* gene regulation.

The entire T3SS cluster consisting of twenty-five genes except one (XAC0395) was up-regulated in XVM2 for both XccA and Xcaw strains. This is consistent with previous report that *Xanthomonas hrp* genes were induced in XVM2 [52,53]. However, only eight *hrp* genes of XccA were reported to be up-regulated by XVM2 in the previous study [52] compared to 24 induced *hrp* genes identified in this study. Among all the effectors, 16 were induced for XccA whereas 19 effectors were overexpressed for Xcaw in XVM2. In the previous study [52], only three effector genes *avrXacE1*, *avrXacE2*, and *Xac0076* of XccA were induced in XVM2. Thus, our study further expanded the knowledge of expression of the *hrp* and effector genes in XVM2 medium.

Interestingly, both *hrpX* and *hrpG* genes were overexpressed in the Xcaw compared to XccA (Additional file 13). Both genes have been shown to be critical for virulence in *Xanthomonas* spp. [54]. The *hrpX* gene encodes an AraC-type transcriptional activator and *hrpG* gene encodes an OmpR family regulator, which are known to regulate many virulence related genes including T3SS effectors, T2SS substrate, flagella, and chemotaxis genes [55]. Overexpression of Xcaw *hrpG* in *X. perforans* elicited HR in grapefruit and Mexican lime leaves probably by inducing *xopA* and other avirulence genes [7]. The *xopA* gene encodes harpin and was suggested to be a host-limiting factor by inducing HR. Its homologues *hpaG* and *hrpN* are also known to induce HR. However, the *xopA* gene was not overexpressed significantly in Xcaw compared to XccA (Additional file 14). The fold change of *xopA* was more than 2, but the FDR did not pass the cut off value. Five other effector genes *xopL*, *xopX*, *xopAD*, *hrpW*, and *xopAQ* were overexpressed in Xcaw in XVM2, whereas only one effector gene *xopAP* was induced in XccA in NB (Additional file 14). Overexpression of those effector genes in Xcaw might contribute to the limited host range of Xcaw. In addition, the *phoP*-*phoQ* two component system genes were overexpressed in Xcaw compared to XccA (Additional file 13). The *phoP* gene encoding a response regulator is predicted to interact with various signal sensor proteins in addition to PhoQ. It is known to activate the response regulator *hrpG* in *X. oryzae* pv. oryzae, thus leading to activation of various virulence and growth factor genes downstream [56]. The *phoQ* gene on the other hand is required for the activity of AvrXA21 in *X. oryzae* pv. oryzae, which determines host-variation of the strain against some rice lines [56]. Thus in Xcaw, overexpression of *phoP-phoQ* could contribute to activation of certain effector genes mentioned above.

T2SS is the major protein secretion system, which secretes toxins and various degradative enzymes to breakdown the cell wall in plant hosts [20]. T2SS and its substrates have been shown to be important for the virulence of XccA [57]. The *xps* genes were down-regulated in XVM2 as compared to in NB for Xcaw with *xpsE* being the most significantly down-regulated (Additional file 10). XpsE is known to be a key component of T2SS, the loss of which leads to lower virulence in *X. oryzae*[4]. For XccA, the *xps* genes were not down-regulated. Down-regulation of *xps* genes in Xcaw but not in XccA might contribute to differences in virulence on different hosts of Xcaw and XccA. In XccA at least 22 genes encoding T2SS substrates were overexpressed as compared to only 12 in Xcaw. On the contrary 11 genes for Xcaw and 8 for XccA were down-regulated. This is similar to the previous study where genes encoding T2SS substrates were found either down-regulated or up-regulated in XVM2 [52]. Specifically, four T2SS substrate protease genes (XAC2537, XAC2763, XAC2999, and XAC4004) were up-regulated in Xcaw compared to XccA in both conditions (Additional file 13). Consequently, Xcaw showed higher protease activity than XccA (Figure 7A). In contrast, multiple cellulase genes (XAC0028, XAC0029, and *engXCA*) were down-regulated in Xcaw compared to XccA (Additional file 13). Pectate lyase gene *pel* (XAC03562) was also down-regulated in Xcaw compared to XccA in NB medium (Additional file 11). Consequently, Xcaw showed lower pectate lyase activity as compared to XccA (Figure 7B). Thus, the protease and pectate lyase activities are consistent with the differential regulation of genes encoding T2SS substrates in Xcaw and XccA.

Compared to Xcaw, multiple virulence genes were overexpressed in XccA which might contribute to its adaption to a broad host range (Additional file 13). These include many reactive oxygen species-scavenging enzyme genes, e.g., *sodC2* and *grpE*, which indicates that XccA might be more adapted to stressful conditions due to the host defense responses of different hosts. Attachment of *Xanthomonas* to plant cell surfaces is important for pathogenicity [58,59]. Multiple genes involved in adherence were overexpressed in XccA in NB medium (Additional file 11) including filamentous haemagglutinin gene *fhaB*, *gum* genes (*gumB* to *gumK*, *gumM*), chemotaxis genes (XAC0611, XAC1666, XAC1891, XAC1893, XAC1894, XAC1895, XAC1896, XAC1897, XAC1899, XAC1900, XAC1902), *mcp* genes (XAC1996, XAC2448, XAC2866, XAC3132), *cheA* (XAC2865), *cheR* (XAC1890), *cheR* (XAC2869), *cheY* (XAC1904) and *cheD* (XAC1889). Multiple transporter genes, which are known to play critical roles in bacteria to acquire nutrients from the intercellular environment, were overexpressed in XccA in XVM2 as compared to Xcaw, e.g., the potassium transporter genes *kdpB*, *kdpC* and *kdpD* and the iron siderophore transporter gene *fhuA* (XAC2185) and XAC2830 (Additional file 12). Altogether, they might contribute to the virulence on broad host of XccA as compared to Xcaw.

## Conclusions

In conclusion, comparative genomic analysis of Xcaw12879, XauB, XauC, and XccA306 provides insights into the virulence mechanism of *X. citri* subsp*. citri.* Our study indicated that AvrGf1 mainly contributes to the host range limitation of Xcaw12879 whereas XopAF contributes to virulence. In addition, we compared the gene expression profiles of XccA306 and Xcaw12879 in NB and XVM2. Our data demonstrated that virulence genes including genes encoding T3SS and its effectors are induced in XVM2 medium. Numerous genes with differential expression in Xcaw12879 and XccA306 were identified. This study lays the foundation to further characterize the mechanisms for virulence and host range of strains of *X. citri* subsp*. citri* and other bacterial pathogens.

## Methods

### Phylogenetic and comparative analysis

The deduced protein sequences of nine housekeeping genes (*uvrD, secA, carA, recA, groEL, dnaK, atpD, gyrB* and *infB*) from 13 completely sequenced and 10 draft *Xanthomonas* spp., and three *Xylella fastidiosa* strains (out-group species) were used to construct the phylogenetic tree. Amino acid sequences were aligned using ClustalW 2.1 [60]. A phylogenetic tree was constructed from the concatenated sequences using CLC Genomics Workbench v6.0 (CLC Bio, Aarhus, Denmark) by the maximum likelihood method. Comparative analyses of XccA306 and Xcaw12879 was conducted by, a two-way BLAST of the nucleotide sequences to identify unique genes in each strain using the standalone blast + software (ncbi-blast-2.2.4). The genes were considered orthologous if reciprocal TBLASTN hits were found between two genes with e-value less than or equal to 10^-10^ and alignments exceeding 60% sequence identity and 60% query gene length. A gene was considered singleton or unique to each strain if it had no hits or with an *e*-values less than or equal to 10^-5^[61,62]. The CRISPRfinder server [63] was used to identify CRISPRs. Only confirmed structures are reported here. Alignment between whole chromosomes was done using the script Promer from the MUMmer package [64]. Promer does alignments between translated nucleotide sequences.

### Preparation of RNA samples for transcriptome analysis

RNA sample preparation and cDNA library generation were performed according to procedures outlined by Filiatrault et al. [65] with some modifications. RNA samples were extracted from XccA306 and Xcaw12879 grown to OD_600_ of 0.4 in XVM2 medium and NB medium at 28°C on shaker at 200 rpm. The starting OD_600_ for each culture was 0.03. Three biological replicates of each strain in each medium were used for RNA extraction. When the OD_560_ reached 0.4 for each condition, RNA was stabilized immediately by mixing 10 ml of the culture with two volumes of RNAprotect bacterial reagent (Qiagen, Valencia, CA). The cells were centrifuged at 5000 × g at 4°C and cell pellets were treated with lysozyme and RNA extractions were performed using RiboPure bacteria kit (Ambion, Austin, TX) per manufacturers” instructions. Genomic DNA was removed by treatment with TURBO DNA-free kit (Ambion, Austin, TX). Total RNA samples were quantified using spectrophotometry (Nanodrop ND-1000, NanoDrop Tech. Inc.). RNA quality was assessed using the Agilent 2100 bioanalyzer (Agilent Technologies, Palo Alto, CA).

### MRNA enrichment and library construction

mRNA was enriched from total RNA using MicrobExpress kit (Ambion) to remove the 23S and 16S ribosomal RNAs (rRNAs). Removal of rRNAs was assessed using an Agilent Bioanalyzer. Double stranded cDNA synthesis was performed following the Illumina mRNA Sequencing sample preparation guide (Cat. No. RS-930-1001) in accordance with the manufacturer’s standard protocol. Enriched mRNA was fragmented via incubation for 5 min at 94°C with the Illumina-supplied fragmentation buffer. The first strand of cDNA was synthesized by reverse transcription using random oligo primers. Second-strand synthesis was conducted by incubation with RNAse H and DNA polymerase I. The resulting dsDNA fragments were further end-repaired, and A-nucleotide overhangs were added. After the ligation of Illumina adaptors, the samples were run on a denaturing gel and the band correlating to 200 (±25) base pairs on the denatured DNA ladder was selected. The selected DNA constructs were amplified by PCR using the primers provided in the Illumina library kit. The amplified constructs were purified and the library was validated using Agilent 2100 bioanalyzer.

### Illumina sequencing and alignment

Paired-end, 75-cycle sequencing of the libraries was performed using an Illumina GAIIx at Yale Center for Genomic Analysis. The raw sequencing reads were further analyzed using CLC Genomics Workbench v6.0 (CLC Bio, Aarhus, Denmark). The reads were trimmed using the quality score limit of 0.08 and maximum limit of 2 ambiguous nucleotides. The trimmed reads were mapped to the genome and the protein-coding genes of XccA306 (GenBank accession no. NC_003919, NC_003921.3 and NC_003922.1) and Xcaw12879, with the parameters allowing mapping of reads to the genome with up to 2 mismatches. The reads mapped to rRNA and the reads not uniquely mapped were removed from further analysis. The expression levels were evaluated by RPKM method as described by Mortazavi et al. [66].

### Differential gene expression analysis

The differential gene expression of the pooled samples from each condition was analyzed using CLC Genomics Workbench v6.0 (CLC Bio, Aarhus, Denmark). RPKM values were normalized using quantile normalization and further log_2_ transformed for statistical analysis. Box plots, hierarchical clustering of samples and principal component analysis were done to examine data quality and comparability. A *t*-test was performed on log_2_-transformed data to identify the genes with significant changes in expression between the two growth conditions and between the two strains. The *p-values* were adjusted for the false discovery rate (FDR) using the Benjamini and Hochberg method [67].

### Quantitative reverse transcription - PCR (qRT-PCR)

To verify the RNA-Seq result, qRT-PCR assays were carried out using the same sets of RNA for RNA-Seq analysis. Gene specific primers listed in Additional file 7 were designed to generate sequences of 100–250 bp in length from the XccA306 genome. qRT-PCR was performed for all 3 biological replicates of XccA306 and Xcaw12879 grown in NB and XVM2 on a 7500 fast real-time PCR system (Applied Biosystems) using QuantiTect™ SYBR® Green RT-PCR kit (Qiagen) following the manufacturers’ instructions. 16S rRNA was used as an endogenous control. The fold change of gene expression was calculated by using the formula 2^-ΔΔC^_T_[68]. The fold change was further log_2_ transformed to compare with the RNA-Seq data.

### Generation of the *xopAF* mutant and *xopAF, avrGf1* double mutant

To construct the *xopAF* deletion mutant, the 1096-bp fragment containing entire *xopAF* gene was amplified using genomic DNA of Xcaw12879 as template and primers xopAFF1 and xopAFR. This resulted in F1, containing a *Bam*HI restriction site within the *xopAF* gene. A 422 bp fragment containing 337 bp of *xopAF* gene and its downstream region was amplified further from F1 using primers xopAFF2-*Bam*HI and xopAFR (Additional file 7), resulting in F2. Both F1 and F2 were digested with *Bam*HI and fragments F3 (414 bp) and F4 (500 bp) were gel purified. The fragments were ligated and cloned into pGEM-T easy vector, resulting in the construct named pGEM-Δ*xopAF* that was confirmed by PCR and sequencing. From pGEM-Δ*xopAF*, an *Apa*I-*Pst*I fragment containing *xopAF* gene with 192 bp internal deletion was transferred into *Apa*I-*Pst*I digested suicide vector pNTPS138, resulting in pNTPS-Δ*xopAF*. The construct pNTPS-Δ*xopAF* was transformed into *E. coli* DH5αλPIR. The construct was purified from *E. coli* and subsequently transferred into Xcaw12879 and Xcaw12879Δ*avrGf1* generated in a previous study [7] by electroporation. Transformants were selected on NA medium supplemented with 50 μg/μl kanamycin. Positive colonies were replicated on both NA plates supplemented with 5% (w/v) sucrose and kanamycin, and only NA and kanamycin. The sucrose sensitive colonies were selected from NA plus kanamycin plate and grown in NB medium overnight at 28°C. The culture was then dilution-plated on NA containing 5% sucrose to select for resolution of the construct by a second cross-over event. The resulting deletion mutant of *xopAF* and double mutant of *xopAF* and *avrGf1* was confirmed by PCR (data not shown). The complete *xopAF* and *avrGf1* genes were complemented in the single and double mutants using pUFR053 and pUFR034 respectively. The resulting complement strains were Xcaw12879Δ*xopAF*-53:*xopAF*, Xcaw12879Δ*avrGf1*-34:*avrGf1* and Xcaw12879Δ*avrGf*Δ*xopAF*-34:*avrGf1*-53:*xopAF* were used in this study.

### Pathogenicity assay

Pathogenicity assays were conducted in a quarantine greenhouse facility at Citrus Research and Education Center, Lake Alfred, FL. XccA306, Xcaw12879, and XcawΔ*avrGf1* strains were grown with shaking overnight at 28°C in NB, centrifuged down and suspended in sterile tap water and the concentrations were adjusted to 10^8^ cfu/ml. The bacterial solutions were infiltrated into fully expanded, immature leaves of Duncan grapefruit, Valencia sweet orange and Hamlin sweet orange, with needleless syringes [54]. The test was repeated three times with similar results. Disease symptoms were photographed 10 days post inoculation.

### Growth assay *in planta*

XccA306, Xcaw12879, XcawΔ*xopAF*, XcawΔ*xopAF*-53:*xopAF,* XcawΔ*avrGf1* and XcawΔ*xopAF*Δ*avrGf1* strains were grown with shaking overnight at 28°C in NB, centrifuged down and suspended in sterile tap water and the concentrations we re adjusted to 10^6^ cfu/ml. The bacterial solutions were infiltrated into fully expanded, immature leaves of Duncan grapefruit, Mexican lime and Valencia sweet orange with needleless syringes [54]. To evaluate the growth of various Xcc strains and mutants in these plants 2 inoculated leaves were collected from each plant at 0, 2, 4, 7, 10, 14 and 21 days. 1 cm^2^ leaf disks from inoculated leaves were cut with a cork borer and then ground in 1 ml sterile water. These were serially diluted and plated on NA plates. The bacterial colonies were counted after 3-day incubation at 28°C. The test was repeated three times independently.

### Pectate lyase and proteinase assay

XccA306 and Xcaw12879 were grown on nutrient agar at 28°C, then suspended in sterile deionized water to the O.D. of 0.3 at 560 nm. Hildebrand’s medium A, B and C were used to test for pectolytic activity [69]. In short the medium contained bromothymol blue dye, calcium chloride, 2% sodium polypectate and 0.4% agar. The pH was adjusted to 4.5, 7.0 and 8.5 for the medium A, B and C. One μl of the cultures were inoculated onto the plates and incubated at 28°C for 6 days before confirming pitting due to pectate lyase production. 10% skim milk agar was used to test the bacterial protease activity. The cultures were grown and suspended in sterile water as explained above. One μl of the cultures were inoculated onto the skim-milk plates and cultured at 28°C for 6 days to observe protease activity.

### Availability of supporting data

The genome sequences of *Xanthomonas citri* subsp. *citri* strain A^w^12879 are available at GenBank under the accession numbers CP003778, CP003779 and CP003780. The RNA-Seq data from this study are available in the NCBI’s Gene Expression Omnibus database under the accession number GSE41519.

## Competing interests

The authors declare that they have no competing financial interests.

## Authors’ contributions

Conceived and designed the experiments: NW and NJ, Performed the experiments: NJ and MOA, Analyzed the data: NJ, NW, DK, FY, JBJ, FFW, JCS and JHG, Wrote the paper: NJ, NW, JBJ, FFW, JCS and JHG. All authors read and approved the final manuscript.

## Supplementary Material

Additional file 1 **Clustered regularly interspaced short palindromic repeats (CRISPRs) in *X. citri* subsp. *citri* str.** A^w^12879 genome predicted using CRISPRfinder. Click here for file

Additional file 2 **Dot-Plot comparison of unique cluster 4 from Xcaw12879 and genome of *X. campestris* pv. campestris strain 8004 done using MUMer.** Red dots represent undisturbed segment conservation whereas blue dots indicate inversion. Click here for file

Additional file 3 **Prediction and comparison of the TAL effector codes encoded by *pthA* genes of *X. citri* subsp. *citri *str.** 306, and *X. citri* subsp. *citri* str. A^w^. Panel A: Prediction of TAL effector codes of PthAw1 and PthAw2. Panel B: The known TAL effector codes of PthA genes from XccA. Panel C: Comparison of the TAL effector codes of PthAw2 and PthA4, homologs in Xcaw12879 and Xcc306 respectively. Click here for file

Additional file 4 Summary of RNA-Seq data of Xcaw12879 and XccA306 in NB and XVM2. Click here for file

Additional file 5 Correlation between biological replicates for RNA-Seq. Click here for file

Additional file 6 **Principal component analysis of DEG of *X. citri* subsp. *citri* str.** 306 (A), and *X. citri* subsp. *citri* str. A^w^ 12879 (W) under NB and XVM2 conditions. Click here for file

Additional file 7 Primers used in this study. Click here for file

Additional file 8 **RNA-seq validation by qRT-PCR.** Comparison of gene expression by qRT-PCR and RNA-seq. The log_2_-fold change of each gene was derived from comparison of either WNB vs ANB or WXVM2 vs AXVM2. The 16S rRNA gene was used as an endogenous control in qRT-PCR. Values of log_2_ fold change are means of three biological replicates. Error bars indicate standard deviation. Blue bars represent values from RNA-seq and yellow bars are values from qRT-PCR. Click here for file

Additional file 9 **Differentially expressed genes of *X. citri *subsp. *citri *str.** 306 (A) in XVM2 medium as compared to NB. Click here for file

Additional file 10 **Differentially expressed genes of *X. citri *subsp. *citri *str.** A^w^12879 (W) in XVM2 medium as compared to NB. Click here for file

Additional file 11 **Differentially expressed genes between *X. citri* subsp. *citri* str.** 306 (A) and *X. citri* subsp. *citri* str. A^w^12879 (W) in NB medium. Click here for file

Additional file 12 **Differentially expressed genes between *X. citri* subsp. *citri* str.** 306 (A) and *X. citri* subsp. *citri* str. A^w^12879 (W) in XVM2 medium. Click here for file

Additional file 13 **Shared differentially expressed genes between *X. citri* subsp. *citri* str.** 306 (A) and *X. citri* subsp. *citri* str. A^w^12879 (W) in both NB medium and XVM2. Click here for file

Additional file 14 **Differential expression of effector genes between *X. citri* subsp. *citri* str.** 306 (A) and *X. citri* subsp. *citri* str. A^w^12879 (W) in both NB medium and XVM2 medium. FDR values are in parenthesis. The effector genes that pass cut-off value of 0.05 are marked in green. Click here for file
