# Supplementary material for: Comparative genomic and transcriptome analyses of pathotypes of Xanthomonas citri subsp. citri provide insights into mechanisms of bacterial virulence and host range
Source: BMC Genomics. 2013 Aug 14;14:551. doi: 10.1186/1471-2164-14-551 (PMC3751643; doi:10.1186/1471-2164-14-551)
Supplement: Additional file 1 — Clustered regularly interspaced short palindromic repeats (CRISPRs) in X. citri subsp. citri str. Aw12879 genome predicted using CRISPRfinder. [file 1471-2164-14-551-S1.docx]

Additional file 1. Clustered regularly interspaced short palindromic repeats (CRISPRs) in *X. citri* subsp. *citri* str. A^w^12879 genome predicted using CRISPRfinder.

| CRISPR | | CRISPR start position | CRISPR end position | CRISPR length | Direct Repeat consensus (length) | Number of Spacers |
| --- | --- | --- | --- | --- | --- | --- |
| Xcaw | 1 | 27400 | 27698 | 298 | TGGAAACGGTCAATGGCAGCATC (23) | 5 |
|  | 2 | 5157634 | 5158851 | 1217 | GTCGCGCCCTCACGGGCGCGTGGATTGAAAC (31) | 18 |
|  | 3 | 5155349 | 5155579 | 230 | GGTCGCGCCCTCACGGGCGCGTGGATTGAAAC (32) | 3 |
| XccA | 1 | 27399 | 27697 | 298 | TGGAAACGGTCAATGGCAGCATC (23) | 5 |
|  | 2 | 4522475 | 4523694 | 1219 | GTCGCGCCCTCACGGGCGCGTGGATTGAAAC (31) | 18 |
