# Supplementary figures and images for: Comparative genomic and transcriptome analyses of pathotypes of Xanthomonas citri subsp. citri provide insights into mechanisms of bacterial virulence and host range
Source: BMC Genomics. 2013 Aug 14;14:551. doi: 10.1186/1471-2164-14-551 (PMC3751643; doi:10.1186/1471-2164-14-551)

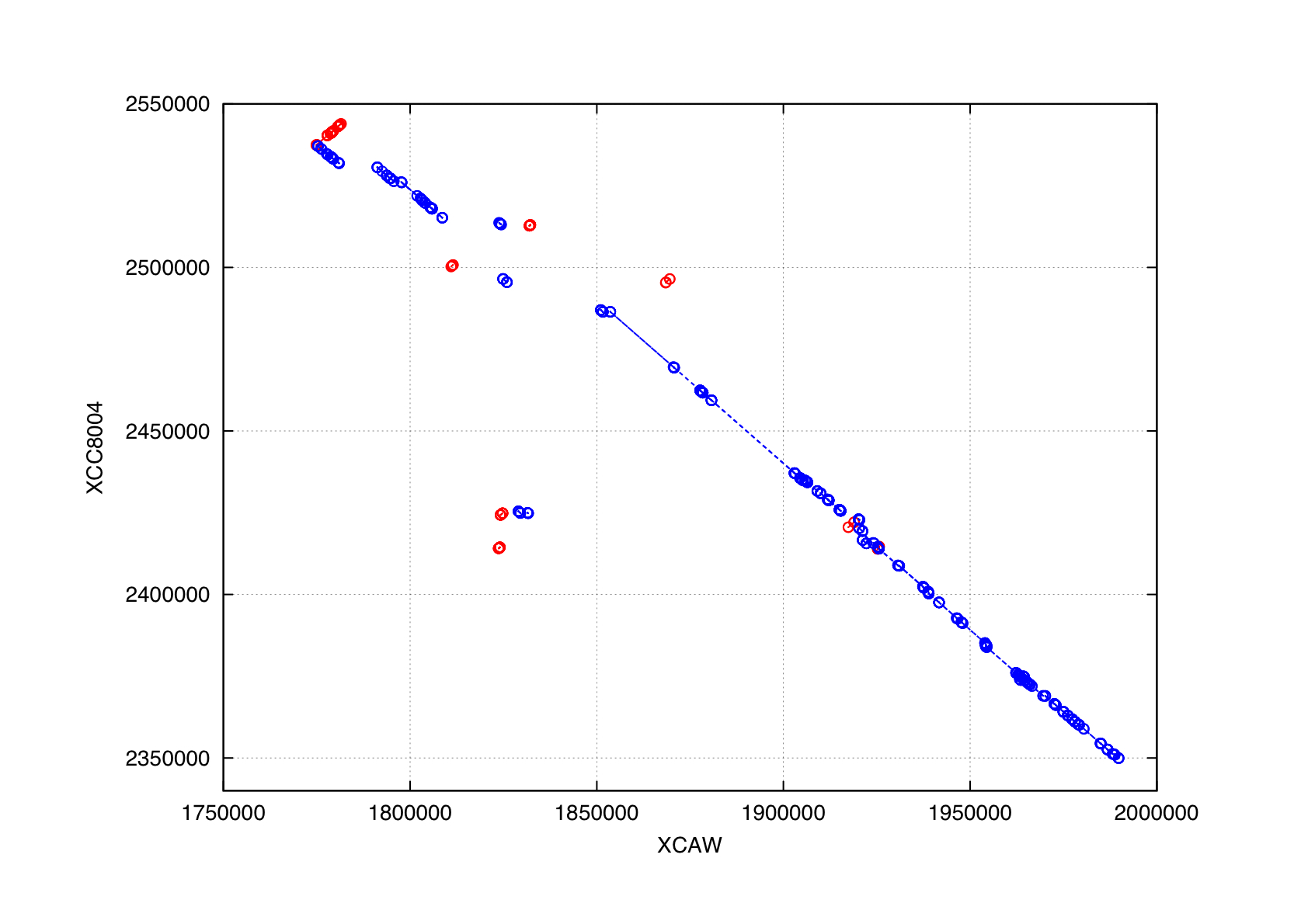

Supplement: Additional file 2 — Dot-Plot comparison of unique cluster 4 from Xcaw12879 and genome of X. campestris pv. campestris strain 8004 done using MUMer. Red dots represent undisturbed segment conservation whereas blue dots indicate inversion. [file 1471-2164-14-551-S2.jpeg]

## Slide 1
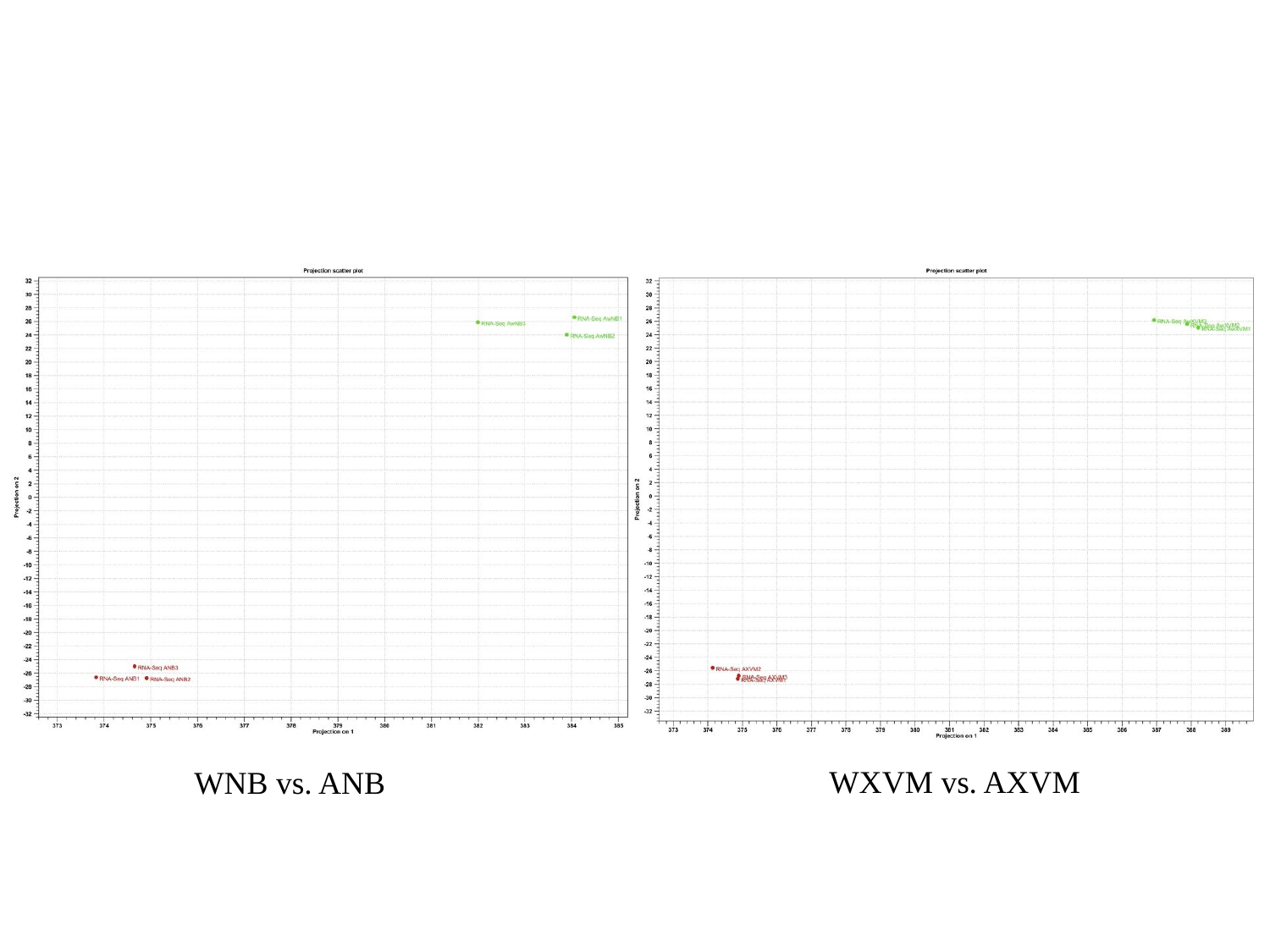

WXVM vs. AXVM
WNB vs. ANB

Supplement: Additional file 6 — Principal component analysis of DEG of X. citri subsp. citri str. 306 (A), and X. citri subsp. citri str. Aw 12879 (W) under NB and XVM2 conditions. [file 1471-2164-14-551-S6.pptx]

## Slide 1
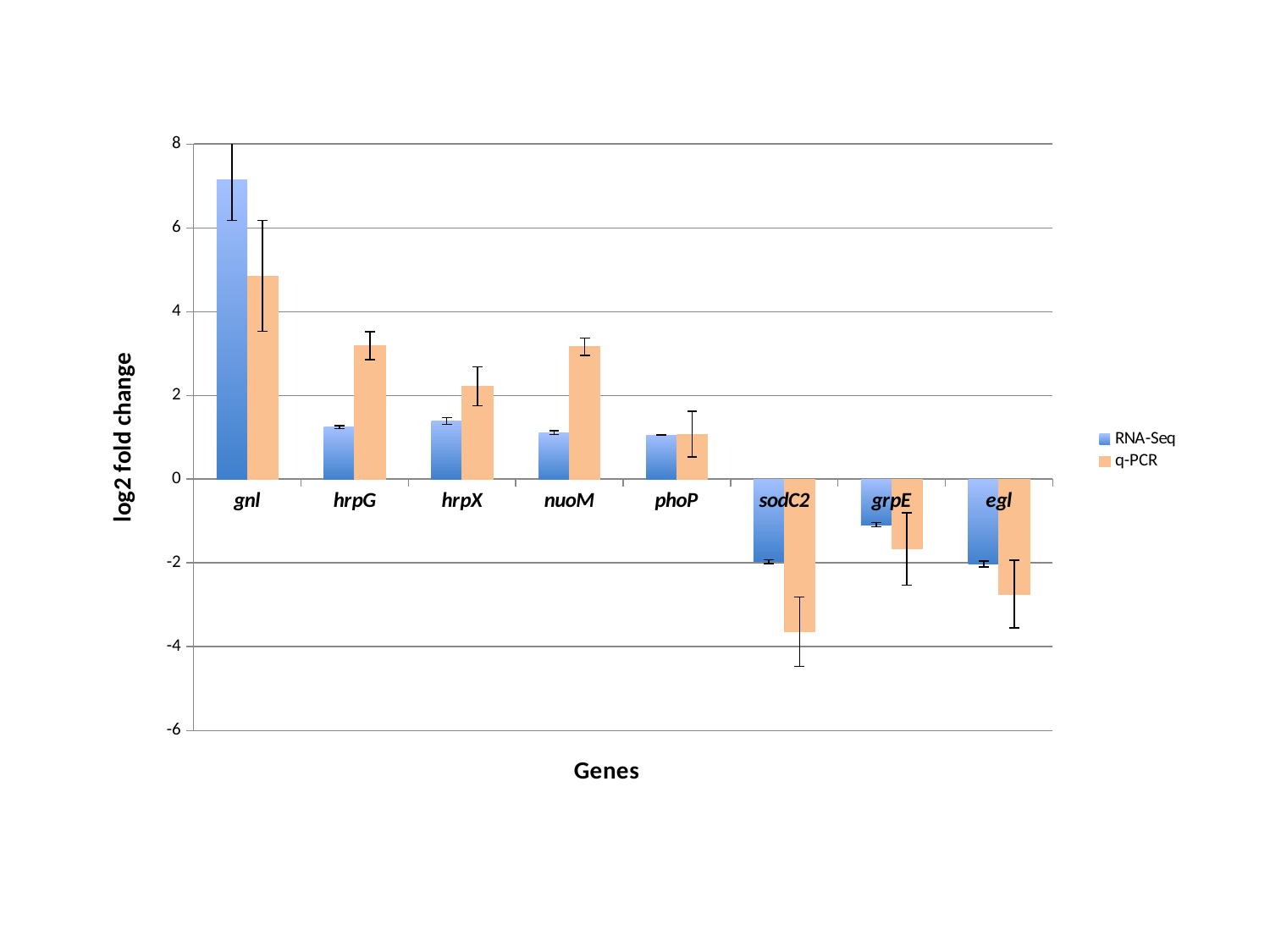

### Chart
| Category | | |
|---|---|---|
| gnl | 7.149999999999999 | 4.85 |
| hrpG | 1.244999999999997 | 3.185 |
| hrpX | 1.39 | 2.215 |
| nuoM | 1.11 | 3.165 |
| phoP | 1.05 | 1.075 |
| sodC2 | -1.970000000000001 | -3.645 |
| grpE | -1.085 | -1.670000000000002 |
| egl | -2.03 | -2.745 |

Supplement: Additional file 8 — RNA-seq validation by qRT-PCR. Comparison of gene expression by qRT-PCR and RNA-seq. The log2-fold change of each gene was derived from comparison of either WNB vs ANB or WXVM2 vs AXVM2. The 16S rRNA gene was used as an endogenous control in qRT-PCR. Values of log2 fold change are means of three biological replicates. Error bars indicate standard deviation. Blue bars represent values from RNA-seq and yellow bars are values from qRT-PCR. [file 1471-2164-14-551-S8.pptx]
