# Supplementary material for: Comparative genomic and transcriptome analyses of pathotypes of Xanthomonas citri subsp. citri provide insights into mechanisms of bacterial virulence and host range
Source: BMC Genomics. 2013 Aug 14;14:551. doi: 10.1186/1471-2164-14-551 (PMC3751643; doi:10.1186/1471-2164-14-551)
Supplement: Additional file 3 — Prediction and comparison of the TAL effector codes encoded by pthA genes of X. citri subsp. citri str. 306, and X. citri subsp. citri str. Aw. Panel A: Prediction of TAL effector codes of PthAw1 and PthAw2. Panel B: The known TAL effector codes of PthA genes from XccA. Panel C: Comparison of the TAL effector codes of PthAw2 and PthA4, homologs in Xcaw12879 and Xcc306 respectively. [file 1471-2164-14-551-S3.pptx]

## Slide 1
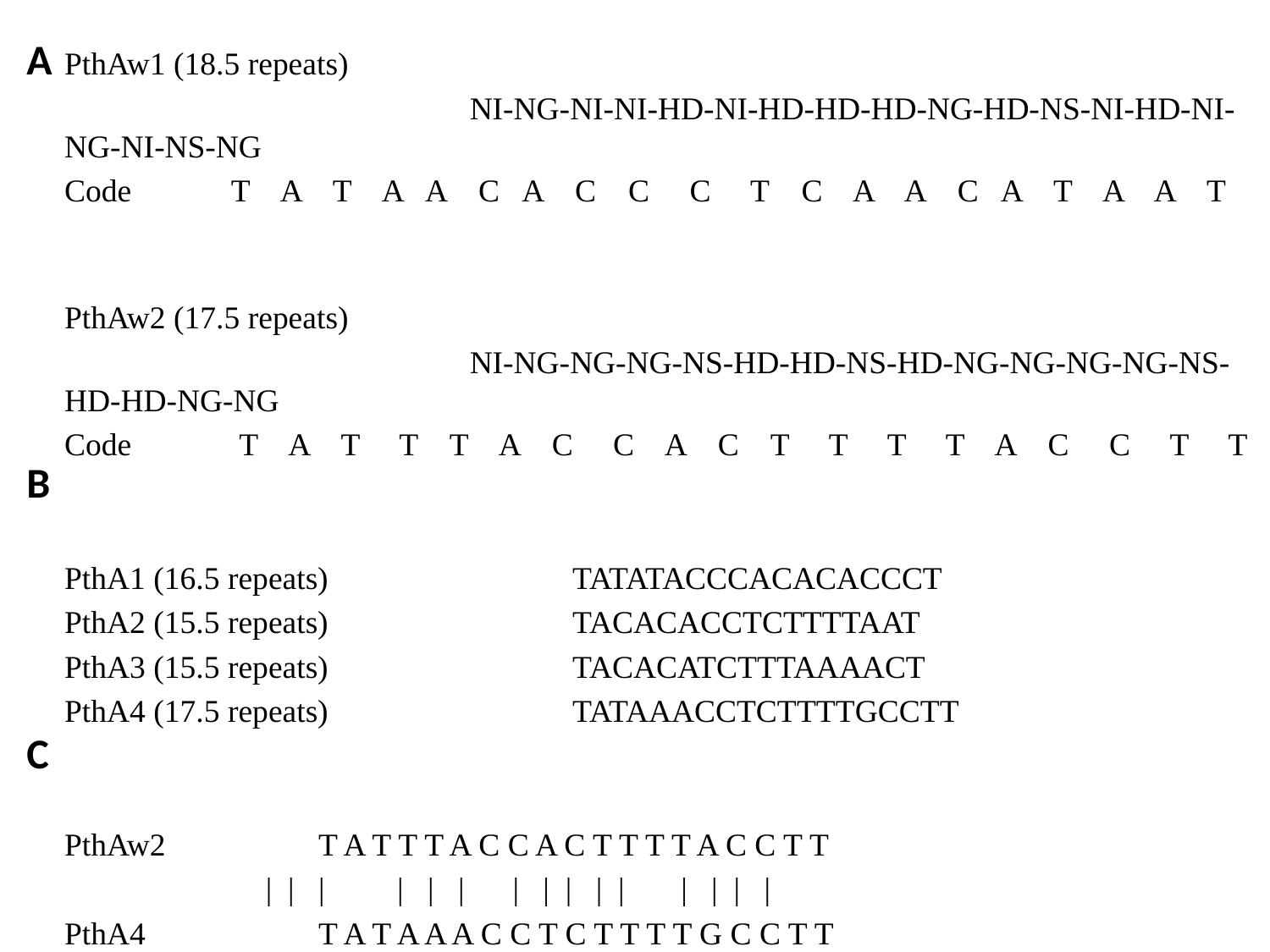

A
B
C
PthAw1 (18.5 repeats)
			 NI-NG-NI-NI-HD-NI-HD-HD-HD-NG-HD-NS-NI-HD-NI-NG-NI-NS-NG
Code	 T A T A A C A C C C T C A A C A T A A T
PthAw2 (17.5 repeats)
			 NI-NG-NG-NG-NS-HD-HD-NS-HD-NG-NG-NG-NG-NS-HD-HD-NG-NG
Code	 T A T T T A C C A C T T T T A C C T T
PthA1 (16.5 repeats)		TATATACCCACACACCCT
PthA2 (15.5 repeats)		TACACACCTCTTTTAAT
PthA3 (15.5 repeats)	 	TACACATCTTTAAAACT
PthA4 (17.5 repeats)		TATAAACCTCTTTTGCCTT
PthAw2 		T A T T T A C C A C T T T T A C C T T
 | | | | | | | | | | | | | | |
PthA4		T A T A A A C C T C T T T T G C C T T
