# Supplementary material for: Comparative genomic and transcriptome analyses of pathotypes of Xanthomonas citri subsp. citri provide insights into mechanisms of bacterial virulence and host range
Source: BMC Genomics. 2013 Aug 14;14:551. doi: 10.1186/1471-2164-14-551 (PMC3751643; doi:10.1186/1471-2164-14-551)
Supplement: Additional file 4 — Summary of RNA-Seq data of Xcaw12879 and XccA306 in NB and XVM2. [file 1471-2164-14-551-S4.docx]

Additional file 4. Summary of RNA-Seq data of Xcaw12879 and XccA306 in NB and XVM2.

| **Sample** | **No. of Reads** | **No. of reads after trim** | **Avg. read length after trim, bp** | **No. of uniquely mapped reads** | **No. of uniquely mapped bps**  **x 10^8^** | **Average**  **coverage** | **mRNA reads % of uniquely mapped reads** | **% of reads mapped to rRNA** |
| --- | --- | --- | --- | --- | --- | --- | --- | --- |
| ANB1 | 61,102,324 | 59,054,234 | 63.8 | 10,900,571 | 6.96 | 147X | 18.46% | 71.66% |
| ANB2 | 34,861,658 | 34,502,250 | 71.0 | 8,505,214 | 6.04 | 129X | 24.65% | 65.12% |
| ANB3 | 55,909,444 | 54,311,690 | 65.5 | 12,850,535 | 8.42 | 179X | 23.66% | 66.58% |
| AXVM1 | 59,932,224 | 57,731,460 | 64.7 | 7,002,216 | 4.53 | 96X | 12.13% | 79.91% |
| AXVM2 | 60,656,960 | 58,303,398 | 62.3 | 8,453,622 | 5.27 | 109X | 14.50% | 76.43% |
| AXVM3 | 59,906,612 | 58,437,562 | 65.8 | 8,721,747 | 5.74 | 120X | 14.93% | 76.52% |
| WNB1 | 64,399,896 | 62,871,274 | 68.3 | 14,093,510 | 9.63 | 202X | 22.42% | 69.74% |
| WNB2 | 23,499,508 | 23,341,184 | 71.9 | 6,671,456 | 4.80 | 101X | 28.58% | 63.98% |
| WNB3 | 52,110,418 | 48,359,192 | 43.0 | 8,185,467 | 3.52 | 77X | 16.93% | 74.54% |
| WXVM1 | 59,681,564 | 58,050,261 | 65.5 | 7,998,317 | 5.24 | 111X | 13.78% | 78.23% |
| WXVM2 | 67,385,040 | 62,470,162 | 61.5 | 7,068,123 | 4.35 | 95X | 11.31% | 80.83% |
| WXVM3 | 60,841,200 | 51,076,418 | 52.0 | 6,561,478 | 3.41 | 79X | 12.85% | 79.26% |

Note: AXVM: XccA306 in XVM2; ANB: XccA306 in NB; WXVM: Xcaw12879 in XVM2; WNB: Xcaw12879 in NB
