# Supplementary material for: Comparative genomic and transcriptome analyses of pathotypes of Xanthomonas citri subsp. citri provide insights into mechanisms of bacterial virulence and host range
Source: BMC Genomics. 2013 Aug 14;14:551. doi: 10.1186/1471-2164-14-551 (PMC3751643; doi:10.1186/1471-2164-14-551)
Supplement: Additional file 5 — Correlation between biological replicates for RNA-Seq. [file 1471-2164-14-551-S5.docx]

Additional file 5. Correlation between biological replicates for RNA-Seq.

| **Sample** | **Repeats compared** | **R^2^ value**  **correlation coefficient** |
| --- | --- | --- |
| XccA306 in NB | ANB1 and ANB2 | 0.99673907 |
|  | ANB2 and ANB3 | 0.996187926 |
|  | ANB3 and ANB1 | 0.996890541 |
| XccA306 inXVM2 | AXVM1 and AXVM2 | 0.999881083 |
|  | AXVM2 and AXVM3 | 0.999844115 |
|  | AXVM3 and AXVM1 | 0.999722292 |
| Xcaw12879 in NB | WNB1 and WNB2 | 0.999145975 |
|  | WNB2 and WNB3 | 0.999762336 |
|  | WNB3 and WNB1 | 0.999658312 |
| Xcaw12879 in XVM2 | WXVM1 and WXVM2 | 0.999227967 |
|  | WXVM2 and WXVM3 | 0.999358198 |
|  | WXVM3 and WXVM1 | 0.999976985 |
