# Supplementary material for: Comparative genomic and transcriptome analyses of pathotypes of Xanthomonas citri subsp. citri provide insights into mechanisms of bacterial virulence and host range
Source: BMC Genomics. 2013 Aug 14;14:551. doi: 10.1186/1471-2164-14-551 (PMC3751643; doi:10.1186/1471-2164-14-551)
Supplement: Additional file 7 — Primers used in this study. [file 1471-2164-14-551-S7.docx]

Additional file 7. Primers used in this study.

| **Primer Sequence 5’ 🡪 3’** |
| --- |
| For Mutant construction  xopAFF1 CGAATCCGAAAAGGCCAT  xopAFF2 GAggatccATTATTACACAGGCGAACG  xopAFR AAGTAGTCGTCTCTGAAAGA  For qRT-PCR   \| gnlF \| TGGATAAATCGCCGGTCAAGGAGT \| \| --- \| --- \| \| gnlR \| ATCGGAGTTGGAGACGTACAAGGT \| \| hrpGF \| ATCGTGCTTGGACGTTTCGATTGC \| \| hrpGR \| ATTGAAAGGCAGCGCAAGGACTTC \| \| hrpXF \| AAGCGTTACTGCTCTACAACCGCT \| \| hrpXR \| TGCGCATTGGTGATCATGTAGCTG \| \| nuoMF \| ACAGGACGACATGAAGAAGCTGGT \| \| nuoMR \| ACGAAACCGTGCGAAATCATCTGC \| \| phoPF \| CTTGCGCGATGAAGGCAAGAAGTT \| \| phoPR \| ACGTGGAACGGCTTGACCAGATAA \| \| sodC2F \| AAGGGTAATGACGTCAAAGGCACG \| \| sodC2R \| ATATTGCCGTGATCGGACTGGGA \| \| grpEF \| GCCTGGACATGACCTACAAGCAAT \| \| grpER \| TTCTGGAACACCTGCACCACAT \| \| eglF \| ACTACGCCAAGTATTACGGCCACA \| \| eglR \| AGGCTCATTCATCAGCCCGAAGAT \| \| 16sF \| AACGCGAAGAACCTTACCTGGTCT \| \| 16sR \| TGCGGGACTTAACCCAACATCTCA \| |
|  |
