# Supplementary material for: Comparative genomic and transcriptome analyses of pathotypes of Xanthomonas citri subsp. citri provide insights into mechanisms of bacterial virulence and host range
Source: BMC Genomics. 2013 Aug 14;14:551. doi: 10.1186/1471-2164-14-551 (PMC3751643; doi:10.1186/1471-2164-14-551)
Supplement: Additional file 14 — Differential expression of effector genes between X. citri subsp. citri str. 306 (A) and X. citri subsp. citri str. Aw12879 (W) in both NB medium and XVM2 medium. FDR values are in parenthesis. The effector genes that pass cut-off value of 0.05 are marked in green. [file 1471-2164-14-551-S14.docx]

Additional file 14. Differential expression of effector genes between *X. citri* subsp. *citri* str. 306 (A) and *X. citri* subsp. *citri* str. A^w^12879 (W) in both NB medium and XVM2 medium. FDR values are in parenthesis. The effector genes that pass cut-off value of 0.05 are marked in green.

| **Effector class** | **Xcaw12879** | **XccA306** | **Log_2_ fold change (FDR)**  **WNB/ANB** | **Log_2_ fold change (FDR)**  **WXVM/AXVM** | **Promoter region** |
| --- | --- | --- | --- | --- | --- |
| AvrBs2 | XCAW_00465 | XAC0076 | 1.04 (0.72) | 1.2 (0.08) | Different -10 |
| XopA (Hpa1/HpaG) | XCAW_00826 | XAC0416 | 1.48 (0.53) | 1.14 (0.16) | Different -35 |
| XopE1 (AvrXacE1) | XCAW_00686 | XAC0286 | -1.03 (0.77) | 1.19 (0.06) | Same |
| XopE3 (AvrXacE2) | XCAW_03515 | XAC3224 | 1.01 (1.0) | 1.04 (0.38) | Same |
| XopF2 | XCAW_01388 Ψ | XAC2785 Ψ | 1.21 (0.21) | 2.39 (0.19) | Same |
| XopI | XCAW_03828 | XAC0754 | 1.74 (0.06) | 1.15 (0.46) | Same |
| XopK | XCAW_03372 | XAC3085 | 1.23 (0.48) | 1.04 (0.83) | Same |
| XopL | XCAW_03376 | XAC3090 | 1.04 (0.82) | 1.59 (0.03) | Same |
| XopQ | XCAW_04706 | XAC4333 | 1.05 (0.72) | -1.07 (0.19) | Same |
| XopR | XCAW_00677 | XAC0277 | -1.01 (1.0) | 1.23 (0.14) | Same |
| XopV | XCAW_03980 | XAC0601 | 1.17 (0.21) | 1.43 (0.08) | Same |
| XopX | XCAW_00956 | XAC0543 | 1.20 (0.20) | 1.20 (0.05) | Same |
| XopZ1 | XCAW_01815 | XAC2009 | 1.03 (0.85) | 1.16 (0.09) | Same |
| XopAD | XCAW_00082 | XAC4213 | 1.03 (0.87) | 1.18 (0.02) | Same |
| XopAI | XCAW_01099 | XAC3230 | -1.02 (0.85) | 1.09 (0.10) | Same |
| XopAK | XCAW_04369 | XAC3666 | -1.01 (0.96) | 1.13 (0.12) | Same |
| XopAP | XCAW_03269 | XAC2990 | -1.07 (0.04) | 1.01 (1.0) | Same |
| HpaA | XCAW_00810 | XAC0400 | -7.31 (0.26) | 1.07 (0.81) | Same |
| HrpW (PopW) | XCAW_03200 | XAC2922 | 1.03 (0.60) | 1.28 (0.02) | Same |
| XopAQ | XCAW_03514 | No annotation between XAC3223 and XAC3224 | -1.02 (0.81) | 1.41 (0.04) | Same |
| XopE2 (AvrXacE3, AvrXccE1) | XCAW_03520 | XACb0011 | -1.00 (1.0) | -1.02 (0.56) | Different -35 |
| XopN | XCAW_01387 | XAC2786 | 1.23 (0.16) | 1.40 (0.06) | Same |
| XopP | XCAW_01310 | XAC1208 | 1.06 (0.36) | 1.16 (0.10) | Same |
| XopAE (HpaF/HpaG) | XCAW_00801 | XAC0393 | -1.10 (0.69) | 1.04 (0.56) | Same |
| XopC2 | XCAW_01311Ψ | XAC1209Ψ  XAC1210Ψ | --  -1.02 (0.99) | --  1.04 (0.64) | Same |
